# Targeted serum proteomics of longitudinal samples from newly diagnosed youth with type 1 diabetes distinguishes markers of disease and C-peptide trajectory

**DOI:** 10.1007/s00125-023-05974-9

**Published:** 2023-08-04

**Authors:** Robert Moulder, Tommi Välikangas, M. Karoliina Hirvonen, Tomi Suomi, Caroline A. Brorsson, Niina Lietzén, Sylvaine F. A. Bruggraber, Lut Overbergh, David B. Dunger, Mark Peakman, Piotr J. Chmura, Soren Brunak, Anke M. Schulte, Chantal Mathieu, Mikael Knip, Laura L. Elo, Riitta Lahesmaa, on behalf of the INNODIA consortium

**Affiliations:** 1 Turku Bioscience Centre, University of Turku and Åbo Akademi University, Turku, Finland; 2 InFLAMES Research Flagship Center, University of Turku, Turku, Finland; 3 Novo Nordisk Foundation Center for Protein Research, Faculty of Health and Medical Sciences, University of Copenhagen, Copenhagen, Denmark; 4 Department of Paediatrics, University of Cambridge, Cambridge, UK; 5 Katholieke Universiteit Leuven/Universitaire Ziekenhuizen, Leuven, Belgium; 6 Immunology & Inflammation Research Therapeutic Area, Sanofi, Boston, MA USA; 7 Sanofi-Aventis Deutschland GmbH, Frankfurt, Germany; 8 Pediatric Research Center, University of Helsinki and Helsinki University Hospital, Helsinki, Finland; 9 Research Program for Clinical and Molecular Metabolism, Faculty of Medicine, University of Helsinki, Helsinki, Finland; 10 Tampere Center for Child Health Research, Tampere University Hospital, Tampere, Finland; 11 Institute of Biomedicine, University of Turku, Turku, Finland

**Keywords:** C-peptide, Glutathione peroxidase 3, Serum proteomics, Type 1 diabetes

## Abstract

**Aims/hypothesis:**

There is a growing need for markers that could help indicate the decline in beta cell function and recognise the need and efficacy of intervention in type 1 diabetes. Measurements of suitably selected serum markers could potentially provide a non-invasive and easily applicable solution to this challenge. Accordingly, we evaluated a broad panel of proteins previously associated with type 1 diabetes in serum from newly diagnosed individuals during the first year from diagnosis. To uncover associations with beta cell function, comparisons were made between these targeted proteomics measurements and changes in fasting C-peptide levels. To further distinguish proteins linked with the disease status, comparisons were made with measurements of the protein targets in age- and sex-matched autoantibody-negative unaffected family members (UFMs).

**Methods:**

Selected reaction monitoring (SRM) mass spectrometry analyses of serum, targeting 85 type 1 diabetes-associated proteins, were made. Sera from individuals diagnosed under 18 years (*n*=86) were drawn within 6 weeks of diagnosis and at 3, 6 and 12 months afterwards (288 samples in total). The SRM data were compared with fasting C-peptide/glucose data, which was interpreted as a measure of beta cell function. The protein data were further compared with cross-sectional SRM measurements from UFMs (*n*=194).

**Results:**

Eleven proteins had statistically significant associations with fasting C-peptide/glucose. Of these, apolipoprotein L1 and glutathione peroxidase 3 (GPX3) displayed the strongest positive and inverse associations, respectively. Changes in GPX3 levels during the first year after diagnosis indicated future fasting C-peptide/glucose levels. In addition, differences in the levels of 13 proteins were observed between the individuals with type 1 diabetes and the matched UFMs. These included GPX3, transthyretin, prothrombin, apolipoprotein C1 and members of the IGF family.

**Conclusions/interpretation:**

The association of several targeted proteins with fasting C-peptide/glucose levels in the first year after diagnosis suggests their connection with the underlying changes accompanying alterations in beta cell function in type 1 diabetes. Moreover, the direction of change in GPX3 during the first year was indicative of subsequent fasting C-peptide/glucose levels, and supports further investigation of this and other serum protein measurements in future studies of beta cell function in type 1 diabetes.

**Graphical Abstract:**

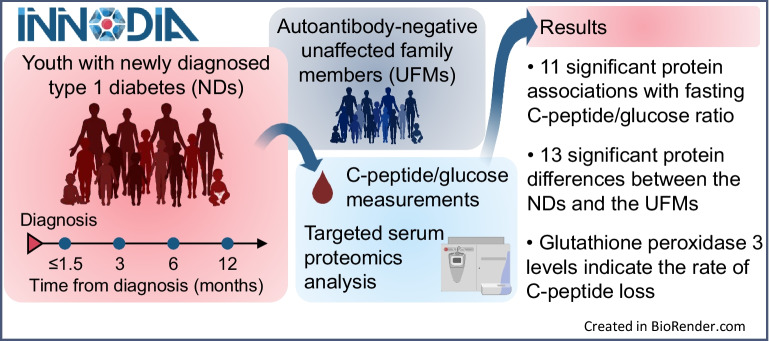

**Supplementary Information:**

The online version contains peer-reviewed but unedited supplementary material available at 10.1007/s00125-023-05974-9.


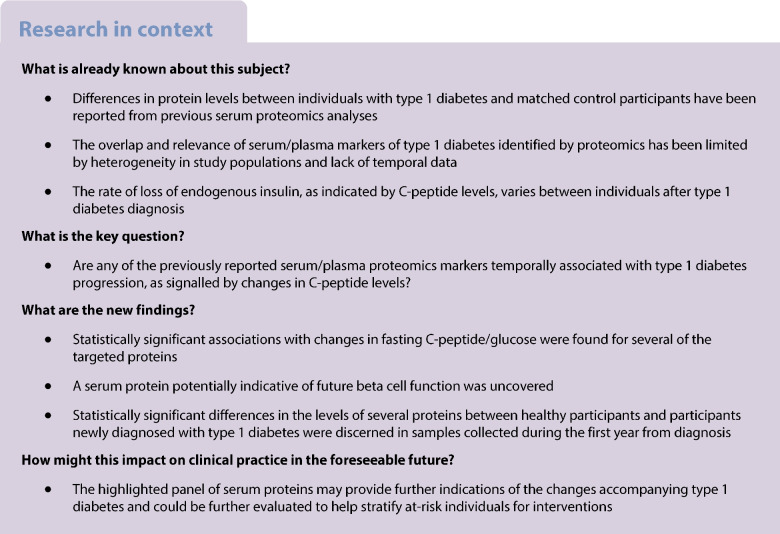


## Introduction

Circulating C-peptide levels, derived from sustained endogenous insulin secretion, provide an indication of preserved beta cell mass, and their decline signifies disease progression in individuals with type 1 diabetes [1]. Changes in other circulating serum proteins could similarly provide clinically relevant information on disease pathogenesis and thus potentially be important for stratification for interventions [2].

Differences in protein abundance have been reported from a range of studies of blood plasma and sera from individuals with type 1 diabetes, before and after diagnosis [3–9]. However, there have been only limited details of temporal changes of these post diagnosis, and with only a small overlap between the different study results [10]. Nevertheless, re-evaluation of these targets with longitudinal measurement and harmonised sample collection might reveal useful associations with changing beta cell function and C-peptide levels. For example, protein level signatures accompanying islet cell destruction or ensuing complications arising from the loss of glycaemic control might be discerned.

To evaluate the utility of type 1 diabetes-associated proteins as signals of changes in beta cell function, targeted MS was used to determine their serum levels in newly diagnosed (ND) youth during the first year from diagnosis. These data were compared with parallel fasting C-peptide/glucose data, and with fasting C-peptide/glucose at 24 months. Additional comparison was made with targeted protein measurements of serum samples from age- and sex-matched autoantibody-negative unaffected family members (UFMs, *n*=194). The measured targets included 85 with a previous type 1 diabetes association, in addition to 13 selected for reference or otherwise. These are summarised, together with the studies from which they were reported and the peptides measured, in electronic supplementary material (ESM) section Protein Selection and ESM Tables 1, 2.

## Methods

### Samples

**Fig. 1:**
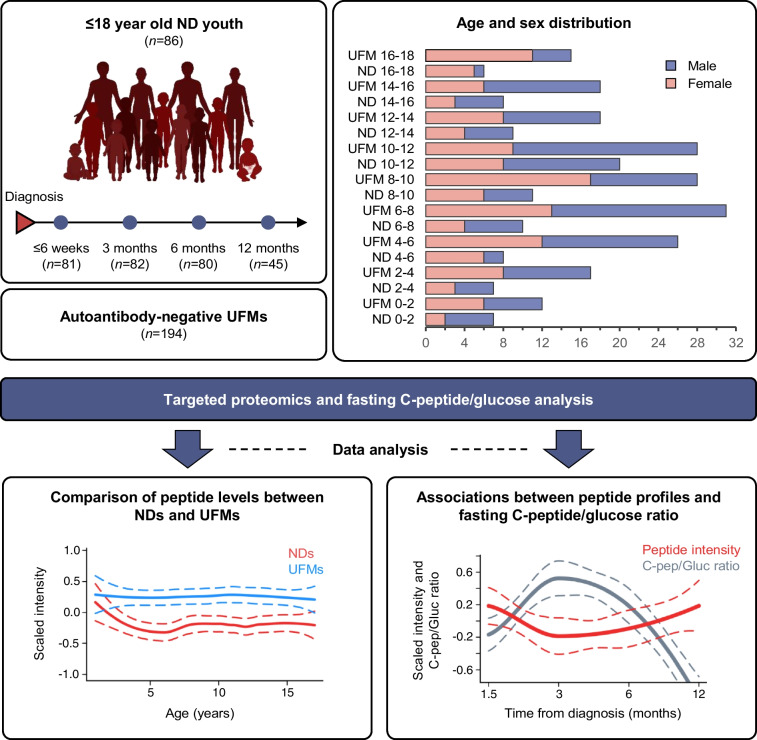
Schematic representation of the study design. Serum samples were collected from ND youth and autoantibody-negative UFMs in the INNODIA type 1 diabetes study. A consecutive recruitment approach was used with inclusion on the basis of an even sex distribution, biosample availability and positivity for at least one diabetes-related autoantibody (GADA, IA-2A, ZnT8A). The age and sex distribution of the cohort is shown in the upper right-hand panel. The samples were prepared and analysed by LC-SRM-MS, targeting a panel of type 1 diabetes-associated proteins. The peptide levels (normalised log_2_-transformed intensity) were compared between ND individuals and UFMs and with fasting C-peptide/glucose (log_*e*_-transformed, pmol/l for fasting C-peptide and mmol/l for fasting glucose) data. BioRender.com was used in creating this figure. C-pep/Gluc, C-peptide/glucose

Serum samples collected between 2018 and 2019 in the pan-European Innovative approaches to understanding and arresting type 1 diabetes (INNODIA) study [11] were used for these measurements. They were selected as an early project milestone to investigate the first 100 ND individuals. Participants between the ages of 1 and 45 years were consecutively recruited on the basis of an even sex distribution and positivity for at least one diabetes-related autoantibody (GADA, IA-2A, ZnT8A). Sex was based on reporting by the parents or the adult study participants, with no selection restrictions applied in terms of regional and socioeconomic factors. The participants were enrolled at the sites of sample collection, as detailed in the ESM INNODIA Project Overview and ESM Table 3. Accurate data on ethnicity were not available, and neither were any specific ethnicity criteria applied. To limit the influence of age, only individuals diagnosed under the age of 18 years (*n*=86, 41 female, 45 male) were considered in the current analysis. In addition, samples from age (±1 year)- and sex-matched autoantibody-negative UFMs (*n*=194, 91 female, 103 male) were analysed. BMI was not used in the criteria for matching. Participant samples were collected within 6 weeks of diagnosis (*n*=81), then at 3 months (*n*=82), 6 months (*n*=80) and 12 months after diagnosis (*n*=45), as detailed in Fig. 1 and ESM Table 3. Only one sample was collected from each UFM

Harmonised protocols for sample collection and storage were used at the study centres. The study followed the guidelines of the Declaration of Helsinki for research on human participants, and the study protocols were approved by the ethical committees of the participating hospitals. Either the parents or participants gave their written informed consent.

### Fasting C-peptide and fasting glucose

Fasting C-peptide and fasting serum glucose were measured at the different visits as previously described [11, 12]. The fasting C-peptide/glucose ratio was used as a surrogate measurement for beta cell function, with a decrease interpreted as an indication of probable disease progression [12].

### Sample preparation and targeted LC-MS/MS

Sera were prepared and analysed by selected reaction monitoring (SRM) with minor modifications to our previously described LC-MS protocol, as detailed in the supplementary information [13]. In brief, serum proteins were alkylated with iodoacetamide, digested with trypsin and spiked with heavy isotope-labelled synthetic peptide analogues (PEPotec, Thermo Fisher Scientific, USA). The samples were prepared batch-wise in 96-well plates. Three distinct quality control (QC) serum samples within each plate were periodically analysed. The plates were designed such that there was no sex bias, and samples from selected sex- and age-matched UFMs were prepared simultaneously to those from ND individuals, in a blinded fashion, and randomised for the order of analysis.

A TSQ Vantage Triple Quadrupole Mass Spectrometer (Thermo Fisher Scientific), coupled with an Easy-nLC 1000 liquid chromatograph (Thermo Fisher Scientific), was used. QC samples were prepared and analysed together with samples and used to assess the system performance and reproducibility.

### Data analysis

#### Pre-processing, normalisation and false discovery rate calculations

Peptide-wise linear mixed effects models (LMMs) were used to normalise the log_2_-transformed data and adjust for batch effects, with consideration of sample analysis order while allowing an individual baseline for each experimental batch. The overall proportion of missing values in the data was low (~0.3%) and no imputation for the missing values was performed. The general technical reproducibility of the normalised and logarithm-transformed data from the QC samples was good, with a mean coefficient of variation of ~3% over all the proteins. Similarly, the Pearson correlation coefficient between the QC samples ranged from 0.9 to 0.999 (with a mean of 0.98), indicating good technical reproducibility, and the pooled median absolute deviation over all the proteins within the QC samples varied between ~0.30 and 0.33, corresponding to the variation observed previously for technical replicate samples in MS experiments [14].

The statistical programming language R version 4.0.0 was used [15], with the R packages lme4 version 1.1–27.1 and lmerTest version 3.1–3 to compose the LMMs [16]. For calculations of peptide intensity differences and associations with beta cell function, a false discovery rate (FDR) of 0.05 was applied after multiple hypothesis correction using the Benjamini–Hochberg procedure.

#### Changing peptide levels and beta cell function

The associations between peptide levels and the ratio of fasting C-peptide/glucose during the first 12 months from diagnosis were investigated as a measure of disease-related beta cell function. Natural logarithm transformation of the fasting C-peptide/glucose ratios was performed to facilitate regression analysis. The LMMs were adjusted for sex, height, BMI score (age-based BMI expressed as standard deviation score [BMI-SDS]), study centre and individual variation. Sex, height and BMI score were included as fixed effects, while individual and study centre were included as random effects, with individual nested under the study centre. As fasting C-peptide is strongly related to body size, height and BMI-SDS were used to control for individual differences in body size while also controlling for possible age-related effects.

To estimate how the peptide levels during the first 12 months were associated with the fasting C-peptide/glucose ratio at 24 months, a linear slope for each peptide and each individual over the first four visits was calculated using linear regression models. LMMs were used to search for associations between the slopes and fasting C-peptide/glucose ratios at 24 months after diagnosis, while adjusting for sex, height, BMI score and study centre as fixed effects and random effects. Additionally, associations were searched for between the peptide slopes and the changes between the fasting C-peptide/glucose ratio at 24 months and the fasting C-peptide/glucose ratio at 6 weeks (24 months/6 weeks). As a criterion for data inclusion, for a slope to be calculated for a peptide for an individual, at least three non-missing values were required for the peptide (out of four possible). Otherwise (less than three non-missing values), the slope for the peptide in question for an individual was set as missing (not available, NA). Also, only ND individuals with at least three visits including the first visit (6 weeks) and a measurement for the fasting C-peptide/glucose ratio at 24 months (*n*=33) were included in the analysis.

#### Differences in the levels of tryptic peptides measured from sera

Peptide-wise LMMs were used to determine significant differences in the levels of peptides from the comparisons of the individuals with type 1 diabetes and the UFMs, while adjusting for age, sex, study centre and individual variation. Age at baseline and sex were included as fixed effects in the LMMs and individual and study centre were included as random effects, with individual nested under the study centre. BMI was not used in this comparison.

## Results

### Longitudinal changes in protein levels associate with changes in fasting C-peptide/glucose

**Fig. 2:**
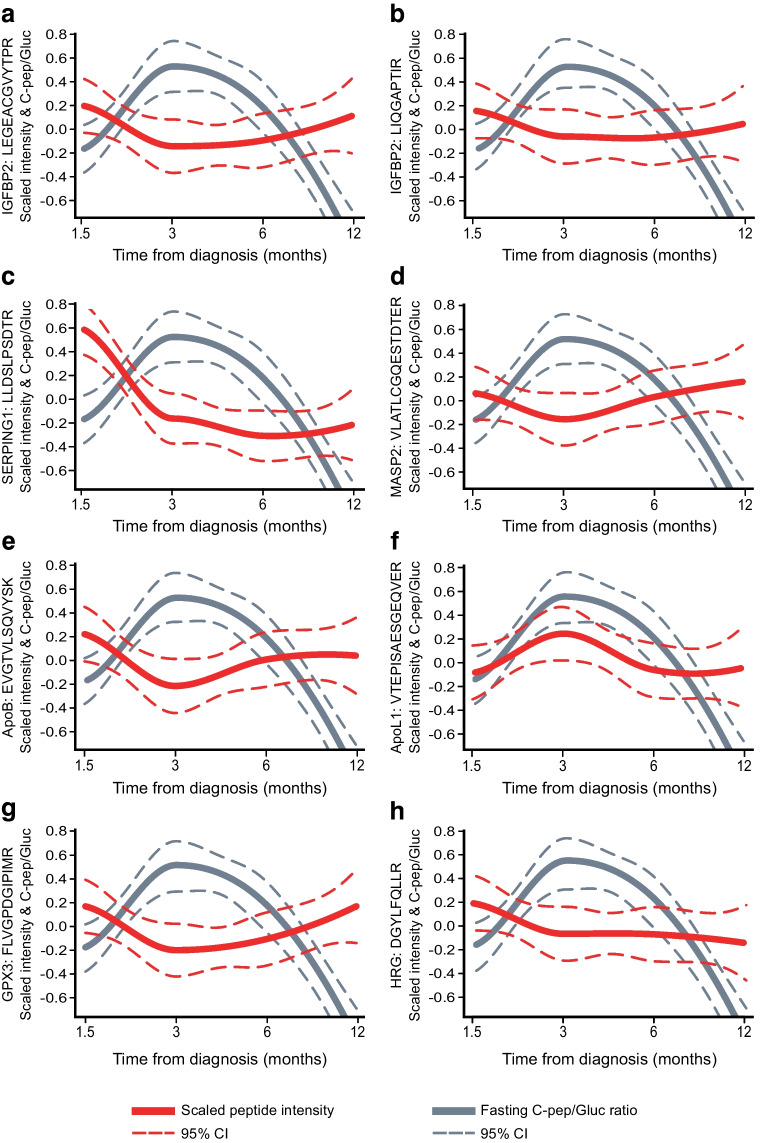
Comparison of the targeted proteomics and fasting C-peptide/glucose data revealed several significant associations (FDR≤0.05). Examples of these are shown for proteins from different classes and processes: IGF proteins (**a**, **b**), coagulation and complement system (**c**, **d**), apolipoproteins (**e**, **f**) and oxidative stress related (**g**, **h**). The data are represented as local regression locally estimated scatterplot smoothing (LOESS) curves for the normalised peptide abundances (red) and fasting C-peptide/glucose (grey) from the ND individuals relative to sampling time from diagnosis. The LOESS curves are represented by the solid lines and their 95% CI by the dashed lines. The peptide and fasting C-peptide/glucose data were adjusted for the potential confounding factors sex, height, standardised BMI, study centre and individual variation. Both the fasting C-peptide/glucose (log_*e*_-transformed, pmol/l for fasting C-peptide and mmol/l for fasting glucose) and peptide expression (normalised log_2_-transformed intensity) data were scaled (*z* score standardised) within each feature for visualisation of both variables during the first year after diagnosis in the same plot. The effect sizes and FDRs for these associations are shown in Table 1. Further representations for these data, including the individual data points, are shown in ESM Fig. 1. C-pep/Gluc, C-peptide/glucose

To determine whether longitudinal changes of the selected proteins were related to beta cell function, the targeted peptide profiles and concurrent fasting C-peptide/glucose ratios were compared over time. In this way, significant associations (FDR≤0.05) were discovered for 12 peptides from 11 proteins (as exemplified in Table 1, Fig. 2 and ESM Fig. 1). Notably, the overall trend in fasting C-peptide/glucose ratios followed a hyperbolic profile during the first year after diagnosis, with only moderate individual variation. Markedly, the most positively associated peptide, apolipoprotein L1 (ApoL1), followed a similar overall profile, whereas the peptide with the strongest inverse association, glutathione peroxidase 3 (GPX3), followed an opposite profile (Table 1, Fig. 2).

**Table 1:** Fasting C-peptide/glucose and SRM peptide associations

Gene	Protein name	Accession number	Peptide	FDR	Effect size^a^
*APOL1*	Apolipoprotein L1	O14791	VTEPISAESGEQVER	0.048	0.51
*IGF1*	Insulin-like growth factor I	P05019	GFYFNKPTGYGSSSR	0.01	0.31
*COL1A1*	Collagen alpha-1(I) chain	P02452	ICVCDNGK	0.035	0.22
*APOB*	Apolipoprotein B-100	P04114	EVGTVLSQVYSK	0.045	−0.22
*IGFBP2*	Insulin-like growth factor-binding protein 2	P18065	LEGEACGVYTPR	0.024	−0.27
*IGFBP2*	Insulin-like growth factor-binding protein 2	P18065	LIQGAPTIR	0.024	−0.28
*MASP2*	Mannan-binding lectin serine protease 2	O00187	VLATLCGCQESTDTER	0.035	−0.36
*HRG*	Histidine-rich glycoprotein	P04196	DGYLFQLLR	0.048	−0.42
*C8G*	Complement component C8 gamma chain	P07360	SLPVSDSVLSGFEQR	0.035	−0.53
*APOM*	Apolipoprotein M	O95445	AFLLTPR	0.048	−0.52
*SERPING1*	Plasma protease C1 inhibitor	P05155	LLDSLPSDTR	0.024	−0.64
*GPX3*	Glutathione peroxidase 3	P22352	FLVGPDGIPIMR	0.003	−1.04

Comparison of fasting C-peptide/glucose data with the targeted MS (SRM) measurements from the individuals with type 1 diabetes revealed 12 significant peptide associations, representing 11 proteins. The FDRs and effect sizes are shown together with the associated peptides

^a^Effect size=LMM regression model coefficient for normalised log_2_-transformed peptide level associations with log_*e*_-transformed fasting C-peptide/glucose (pmol/l for fasting C-peptide and mmol/l for fasting glucose)

**Fig. 3:**
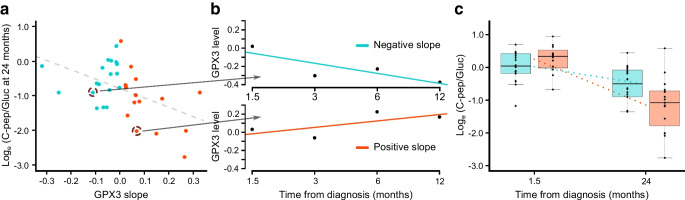
Changes in GPX3 levels during the first year after diagnosis are associated with beta cell function at 24 months. (**a**) Fasting C-peptide/glucose ratio at the 24 month visit vs GPX3 slope during the first 12 months after diagnosis. Individuals with negative or positive slopes are shown in blue and red dots, respectively. The dashed grey line indicates the overall negative association. (**b**) Examples of GPX3 changes during the first year after disease onset for two individuals. The solid lines show a negative slope (blue) and a positive slope (red), with the associated fasting C-peptide/glucose data indicated by the arrows from panel (**a**). The GPX3 data points are marked in black. (**c**) Fasting C-peptide/glucose was better preserved in individuals whose GPX3 levels decreased in the first year from diagnosis (FDR=0.07). The 1.5 months (6 weeks) and 24 months fasting C-peptide/glucose (log_*e*_-transformed, pmol/l for fasting C-peptide and mmol/l for fasting glucose) data are shown for individuals with increasing and decreasing GPX3 levels (normalised log_2_-transformed intensity) in the red and blue boxplots, respectively. The box plots indicate the first, second and third quartiles, and the whiskers show the smallest and largest values within 1.5 × the interquartile range, for the lower and upper limit, respectively. The GPX3 slopes and the fasting C-peptide/glucose ratio data were adjusted for the potential confounding factors sex, height, standardised BMI, study centre and individual variation. The fasting C-peptide/glucose data were natural log transformed. C-pep/Gluc, C-peptide/glucose

To further determine if the early changes in peptide/protein abundance are related to the change in C-peptide in the longer term, the trends in peptide levels during the first year were compared with the fasting C-peptide/glucose ratios at 24 months. Although this comparison was made for all the targets, only the changes for the GPX3 peptide were indicative of a future decline in fasting C-peptide levels and thus beta cell function (FDR≤0.05, Fig. 3a–c). This association remained strong even when comparing trends in peptide levels during the first year with changes in the fasting C-peptide/glucose ratio between the baseline 6 week and 24 month measurements (FDR=0.07). The preservation of C-peptide was favoured in participants with decreasing GPX3, whereas the loss of C-peptide was more pronounced for those with increasing GPX3 (Fig. 3a–c).

### Comparison of the ND individuals and UFMs demonstrates differences in the peptide levels during the first year after diagnosis

**Fig. 4:**
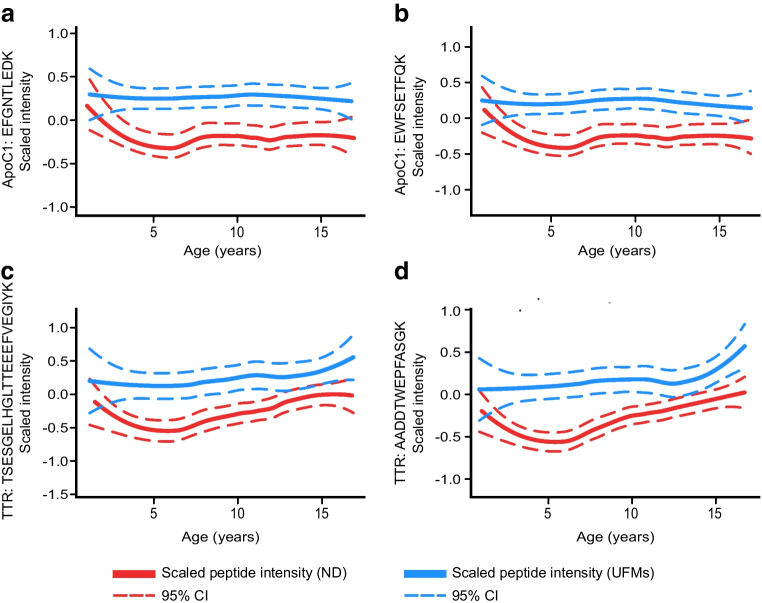
Comparison of the targeted proteomics data from the age-matched ND individuals and UFMs revealed significant differences for a series of peptides (FDR≤0.05). Examples are shown for peptides from ApoC1 (**a**, **b**) and TTR (**c**, **d**). The data are shown as the locally estimated scatterplot smoothing (LOESS) curves for the peptide abundances (normalised log_2_-transformed intensity) relative to age and grouped according to status, i.e. ND individuals (red) and UFMs (blue). The LOESS curves are represented by the solid lines and their 95% CI by the dashed lines. The effect sizes and FDRs for these differences are shown in Table 1. Further representations for these data, including the individual data points, are shown in ESM Fig. 2

Since the selected targets were chosen from a range of different experimental settings, comparisons between these ND youth and matched UFMs were made to evaluate their status in the present cohort. This highlighted significant differences in the levels of 18 peptides, representing 13 proteins (FDR≤0.05, Table 2). Notably, there were consistent and significant differences for multiple peptides from transthyretin (TTR) (Fig. 4c, d), apolipoprotein C1 (ApoC1) (Fig. 4a), complement 2 and afamin (AFM) (ESM Fig. 2, Table 2). Other differences included peptides from the IGF family proteins, IGF1 and IGF-binding proteins 1 and 3 (IGFBP1 and IGFBP3). BMI was not considered for these comparisons.

**Table 2:** Peptides differentially abundant in the comparison of ND individuals and UFMs

Gene	Protein	Accession number	Peptide	FDR	Effect size^a^
*HBB*	Haemoglobin subunit beta	P68871	SAVTALWGK	4.7 × 10^−2^	0.4
*IGFBP1*	Insulin-like growth factor-binding protein 1	P08833	AQETSGEEISK	8.0 × 10^−6^	0.38
*F2*	Prothrombin	P00734	TATSEYQTFFNPR	8.5 × 10^−9^	0.26
*HRG*	Histidine-rich glycoprotein	P04196	DGYLFQLLR	1.7 × 10^−2^	0.16
*HGFAC*	Hepatocyte growth factor activator	Q04756	LEACESLTR	1.3 × 10^−2^	0.14
*C2*	Complement 2	P06681	AVISPGFDVFAK	1.2 × 10^−5^	0.13
*C2*	Complement 2	P06681	HAFILQDTK	3.9 × 10^−2^	0.08
*GPX3*	Glutathione peroxidase 3	P22352	FLVGPDGIPIMR	5.5 × 10^−3^	0.11
*IGFBP3*	Insulin-like growth factor-binding protein 3	P17936	ETEYGPCR	3.0 × 10^−2^	−0.11
*AFM*	Afamin	P43652	DADPDTFFAK	5.5 × 10^−4^	−0.13
*AFM*	Afamin	P43652	AESPEVCFNEESPK	7.1 × 10^−5^	−0.16
*AFM*	Afamin	P43652	GQCIINSNK	2.6 × 10^−4^	−0.2
*TGFBI*	TGF-β-induced protein ig-h3	Q15582	LTLLAPLNSVFK	4.0 × 10^−4^	−0.22
*IGF1*	Insulin-like growth factor I	P05019	APQTGIVDECCFR	3.8 × 10^−2^	−0.35
*APOC1*	Apolipoprotein C1	P02654	EFGNTLEDK	8.5 × 10^−09^	−0.42
*APOC1*	Apolipoprotein C1	P02654	EWFSETFQK	1.2 × 10^−7^	−0.46
*TTR*	Transthyretin	P02766	AADDTWEPFASGK	8.5 × 10^−9^	−0.44
*TTR*	Transthyretin	P02766	TSESGELHGLTTEEEFVEGIYK	4.2 × 10^−7^	−0.46

Analysis of the SRM data revealed significant differences for 18 peptides from 13 proteins in the comparison between ND individuals and UFMs. These results are displayed with the FDRs and effect sizes. The effect sizes were derived from the LMMs with adjustment made for confounding effects to detect statistically significant differences

^a^Effect size=LMM regression model coefficient related to the change in normalised log_2_-transformed peptide intensity level and the condition status (ND vs UFM)

## Discussion

Targeted proteomics was used to evaluate potential markers of type 1 diabetes in youth from the first 100 ND individuals recruited to the INNODIA study. The data revealed associations with fasting C-peptide/glucose changes and differences between the UFMs and ND youth for a panel of these proteins. The fasting C-peptide/glucose data followed a parabolic profile, reflective of the so-called honeymoon period that has been reported in type 1 diabetes [17]. In this way, the concordant changes in the measured proteins could provide insights into the disease pathology. For conciseness and clarity of discussion, the clearest examples of fasting C-peptide/glucose-associated and differentially abundant proteins are presented and subdivided by their common biological processes and connecting themes.

### Markers of oxidative stress and declining C-peptide levels

The strongest inverse association with the fasting C-peptide/glucose ratio was observed for the peptide measured for GPX3. Accordingly, the level of GPX3 declined as the fasting C-peptide/glucose increased and vice versa. Furthermore, a comparison with 24 month fasting C-peptide/glucose revealed that the rate of change in GPX3 during the first year after diagnosis gave an indication of the loss of C-peptide by 24 months (Fig. 3). Notably, the GPX3 peptide was also significantly more abundant in the type 1 diabetes group (Table 1).

GPX3 is a selenocysteine-containing protein and a major scavenger of reactive oxygen species (ROS) in serum [18]. Its expression is directly affected by selenium (Se) abundance, i.e. sensitive to decreasing Se levels, and it is transcriptionally regulated by peroxisome proliferator-activated receptor γ (PPARγ) [19]. GPX3 protects cells and enzymes from oxidative damage by catalysing the glutathione (GSH) reduction of hydroperoxides [18], and its activity is thereby limited by the availability of serum GSH, although not by Se. In keeping with the role of GPX3 as a stress-responsive antioxidant enzyme, there are binding sites at its promoter for hypoxia-inducible factor 1 (HIF-1), specificity protein 1 (Sp1) transcription factor, a metal response element (MRE) and an antioxidant response element (ARE) [20].

The observed difference between the individuals with type 1 diabetes and UFMs (Table 1) was in contrast to the cross-sectional serum proteomics study of Zhang et al, in which the authors reported downregulation of GPX3 in type 1 diabetes and type 2 diabetes [4]. There are few other clear human data on serum GPX3 levels in type 1 diabetes, while there are several reports on type 2 diabetes with other comorbidities [21–23], including suggestions that the serum/plasma levels are related to oxidative stress and dependent on the extent of disease progression [24]. For example, higher GPX3 and PPARγ expression has been reported during disease onset, with a subsequent decrease with advancing disease [19, 21]. In addition, positive and inverse associations have been reported for the activity and expression of GPX3 in other diseases [24]. In Crohn’s disease, for example, where oxidative stress leads to mucosal layer damage, glutathione peroxidase (GPx) activity is increased during the active phase and returns to normal during remission [25]. The association observed with GPX3 could similarly reflect changes in oxidative stress, where the decrease in GPX3 is in parallel with gains in endogenous insulin secretion and improved glycaemic control. Alternatively, these observations could be related to an underlying immune response [26].

Histidine-rich glycoprotein (HRG) was also inversely associated with the fasting C-peptide/glucose ratio and more abundant in ND individuals compared with UFMs. Notably, in relation to GPX3, HRG–GPx binding and enhancement of GPx activity, including GPX3, was recently reported [27].

Also, in the context of oxidative stress, the vitamin E binding protein AFM was less abundant in the individuals with type 1 diabetes than in the UFMs. Reduced vitamin E levels have been reported in studies on individuals affected by type 1 diabetes, and supplementation of vitamin E has been used to reduce oxidative stress [28].

### Changes in the fasting C-peptide/glucose ratio and apolipoproteins

The strongest positive association with changes in fasting C-peptide/glucose was observed for ApoL1. Notably, type 1 diabetes is accompanied by disorders in lipid metabolism, including alterations in lipoprotein metabolism where insulin plays a central regulatory role [29]. ApoL1 positively correlates with plasma triacylglycerol levels in both healthy and diseased conditions [30]. In a study of type 2 diabetic individuals, low C-peptide levels were associated with reduced ApoL1, and insulin was shown to increase ApoL1 secretion in hepatic cells [30]. The positive association of ApoL1 with the fasting C-peptide/glucose ratio in the current study may similarly indicate the effect of changes in residual insulin secretion and ApoL1 levels.

An inverse association with the fasting C-peptide/glucose ratio was observed for one of the apolipoprotein B-100 (ApoB) peptides. Insulin suppresses hepatic ApoB secretion and increases the clearance of ApoB-containing lipoprotein particles, which include VLDLs, intermediate-density lipoproteins, LDLs and chylomicrons [31]. Insulin deficiency is associated with accumulation of VLDL and chylomicrons leading to hypertriglyceridaemia, which is reversible with adequate insulin therapy [32]. In this manner, the observed relationship might reflect how sustained endogenous insulin affects ApoB secretion and/or ApoB-containing particle clearance.

Lower levels of ApoC1 were detected in the ND individuals than in the UFMs. ApoC1 is exchangeable between different lipoprotein classes and modulates a range of enzymes important in lipid metabolism, including cholesteryl ester transfer protein (CETP), which transfers cholesteryl esters and triacylglycerols between lipoproteins [33]. Increased CETP activity was noted in individuals with type 1 diabetes, resulting in peripheral hyperinsulinaemia [29]. Although subcutaneous insulin administration could cause the enhanced CETP activity in the latter example [34], our results suggest that the lower level of CETP-inhibiting ApoC1, observed in the ND individuals, may also play a role. Post-translational modifications of ApoC1 impair its ability to inhibit CETP [35]. In addition, levels of ApoC1 are reduced at the onset of type 1 diabetes [3], and further studies are needed to explore the role of ApoC1 in the development of type 1 diabetes.

Taken together, these results suggest that the profiles of these apolipoproteins and their relationships with fasting C-peptide/glucose ratio should be taken into further consideration for monitoring changes associated with type 1 diabetes.

### ND individuals show alterations in serum IGF proteins

Lower IGF1 and IGFBP3 and elevated IGFBP1 levels were detected in the sera of ND youth, compared with UFMs. As noted in the literature when selecting these targets, reduced levels of IGF1 have been reported in children, adolescents and adults who are autoantibody positive (AAb+) or who have type 1 diabetes [36]. Similarly, the positive association between IGF1 and the fasting C-peptide/glucose ratio follows reported positive correlations of C-peptide and IGF1 levels in AAb+ individuals and in those with type 1 diabetes [36, 37]. In keeping with this trend, serum IGF1 has been reported to increase in ND individuals after the initiation of insulin treatment [38]. Overall, these observations are consistent with the requirement for sufficient insulin to maintain normal hepatic IGF1 production, and the challenges to maintain exogenous insulin administration.

Previous studies of individuals affected by type 1 diabetes [37, 38] and AAb+ individuals [36] have revealed increased serum IGFBP1 levels. IGFBP1 expression is dependent on insulin, which downregulates its production. In keeping with an anticipated challenge to endogenous insulin secretion, elevated IGFBP1 levels were detected in the ND group. Further, in relation to the detection of lower participant levels of IGF1, IGFBP1 modulates IGF1 bioavailability, such that elevated IGFBP1 is limiting [39].

Finally, the two peptides measured for IGF-binding protein 2 (IGFBP2) were both inversely associated with the fasting C-peptide/glucose ratio. In human studies, IGFBP2 has been reported to correlate inversely with insulin levels [40], and in the cross-sectional serum proteomics study by Zhi and co-workers, elevated IGFBP2 was observed and validated in individuals with type 1 diabetes [5]. Although significant differences in protein levels were not found between the UFMs in the current analyses, the observation from the larger validation cohort measured by Zhi et al (1139 type 1 diabetic individuals and 848 control individuals) may have benefited from the improved statistical power. In contrast to the ND individuals, in longitudinal serum and plasma proteomics studies, lower and decreasing levels of IGFBP2 were reported prior to the clinical manifestation of type 1 diabetes [6, 8]. The latter differences are potentially a result of the developing prediabetic autoimmune condition, rather than directly insulin related.

### Increased serum coagulation factors and type 1 diabetes

Prothrombin (F2) was more abundant in the ND individuals relative to the UFMs, and was similarly reported in the serum proteomics study by do Nascimento de Oliveira et al [9]. F2 is an integral component of the clotting process and other studies of both type 1 and type 2 diabetic individuals have reported high coagulation factor levels, including F2 [41]. Consistent with the theme of coagulation, an inverse association with fasting C-peptide/glucose was observed for a peptide from plasma protease C1 inhibitor (SERPING1). SERPING1 is important in blood coagulation and regulation of complement activation. In the cross-sectional serum proteomics study by Zhang et al, higher serum levels of SERPING1 provided excellent sensitivity and specificity, distinguishing type 1 diabetic individuals and matched control individuals [4]. Although no significant difference in the SERPING1 levels relative to the UFMs was detected in the current study, its inverse association with fasting C-peptide/glucose further suggests its relationship with beta cell function in type 1 diabetes. Hepatocyte growth factor activator (HGFAC) is activated downstream of the coagulation cascade and was more abundant in the ND individuals. Finally, MBL associated serine protease 2 (MASP2) (inversely correlated with fasting C-peptide/glucose) is involved in activating the complement system, and its increased abundance was putatively linked to blood glucose levels in type 1 diabetic individuals [42]. Collectively, this representation of coagulant and complement proteins could embody the development of an enhanced thrombotic environment that has been observed with long-term diabetes [42]. It should be noted that BMI was not considered for the UFM vs ND comparisons.

### Differences in proteins associated with islet survival and beta cell integrity

In agreement with the observations from the cross-sectional serum proteomics study of Zhi and co-workers [5], TGF-β-induced protein ig-h3 (TGFBI) was less abundant in the individuals with type 1 diabetes. Notably, human genome-wide association study (GWAS) data have shown that single-nucleotide polymorphisms in the vicinity of the *TGFBI* gene are associated with type 1 diabetes risk, and TGFBI has been shown to promote islet survival, function and regeneration in mice [43]. TTR was also detected at lower levels in the individuals with type 1 diabetes, and, of further relevance to the islet cells, the homo-tetramer of TTR has been reported in relation to the preservation beta cell integrity and secretion [44]. In the serum proteomics study of Zhang et al*,* lower serum levels of TTR were observed in both type 1 and type 2 diabetic individuals [4]. These observations for TGFBI and TTR suggest that lower circulating levels of these islet-associated proteins could reflect changes in beta cell function accompanying type 1 diabetes.

### Strengths and limitations of the study

This study benefited from several strengths in its design and sample collection. First, the longitudinal data and repeated samples from the same individuals increased the statistical power. Similarly, the early collection of samples from ND individuals enabled analysis of the initial signs of disease and associated changes in beta cell function. Additionally, the availability of samples from age-matched UFMs provided a cross-reference of the healthy state, even though BMI was not considered for the comparisons. Technically, the use of a targeted proteomics approach provided higher selectivity and sensitivity than a discovery proteomics approach. This also removed the need to deplete high-abundance proteins, saving time and lessening the influence of technical variation. Despite these benefits, the targeted approach was limited by the preselection of expected markers and the numbers of their associated peptides. Nevertheless, statistically significant associations with fasting C-peptide/glucose and differences between the levels of the targets were detected. In future studies, however, these findings should be further validated in independent cohorts and compared with other existing potential serum protein markers of type 1 diabetes.

Despite the strengths provided through the INNODIA study design, the representation of different centres, spread of age, autoantibody combinations and limited number of individuals with 12 month samples could have masked some underlying differences. The unbiased, consecutive recruitment may have introduced different disease endotypes or subtypes. Of note, one ND individual was later identified to have maturity-onset diabetes of the young; however, this is unlikely to impact the results of the study. As mixed meal tolerance tests (MMTTs) were not performed for participants of 5 years of age or younger or at 6 weeks from diagnosis, fasting C-peptide/glucose was used rather than MMTT AUC due to larger coverage of the time series. Overall, fasting C-peptide/glucose provides a similar representation of beta cell function to MMTT AUC [12], and, in line with earlier reports, we detected a very strong concordance between fasting C-peptide/glucose and MMTT AUC (ESM Figs. 3, 4).

### Summary

**Fig. 5:**
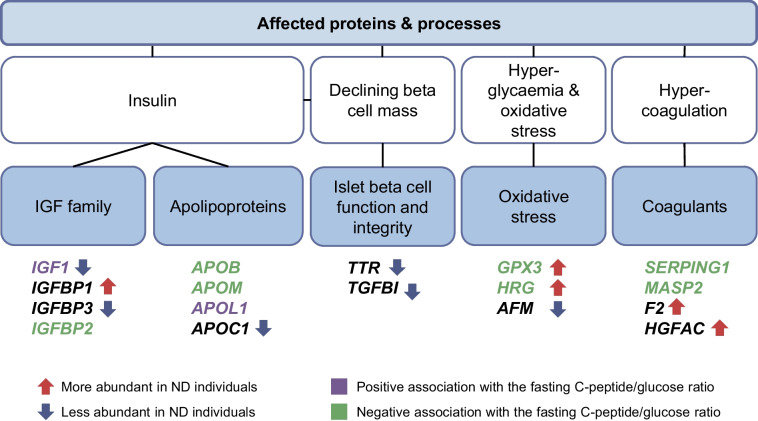
Pathways and processes highlighted by targeted serum proteomics analysis of longitudinal serum samples from ND individuals. The proteins associated with fasting C-peptide/glucose and/or detected at different levels (relative to the UFMs) included a range of apolipoproteins, coagulants, IGF family members and proteins involved with oxidative stress and beta cell function and integrity. These are represented with the gene names coloured according to the fasting C-peptide/glucose association (mauve and green, positive and inverse, respectively), with upward and downward coloured arrows to indicate the difference in levels relative to age- and sex-matched UFMs (red, greater; blue, lower)

Overall, pathways, processes and protein classes linked to the pathogenesis of type 1 diabetes were reiterated in the findings from this study, as summarised in Fig. 5. Examples include apolipoproteins and insulin-associated IGFs and their binding proteins. Likewise, the differences observed for GPX3 and HRG could stem from the challenges created by hyperglycaemia. Furthermore, the differences in F2 and HGFAC, together with associations related to MASP2 and SERPING1, suggest the hypercoagulative state previously reported in type 1 diabetes [41]. These results suggest that the dysregulation of proteins and their related pathways is already apparent in the first year of disease. The relationships between these changes and different rates of decline of fasting C-peptide/glucose suggest both a basis for the stratification of individuals with type 1 diabetes and a window for intervention.

### Supplementary Information

ESM 1 (PDF 1.81 MB)

## Data Availability

Access to these person-sensitive data is only through secure environments and by application to the INNODIA Data Access Committee.

## Abbreviations

AAb+: Autoantibody positive
AFM: Afamin
ApoB: Apolipoprotein B-100
ApoC1: Apolipoprotein C1
ApoL1: Apolipoprotein L1
BMI-SDS: BMI expressed as standard deviation score
CETP: Cholesteryl ester transfer protein
F2: Prothrombin
FDR: False discovery rate
GPx: Glutathione peroxidase
GPX3: Glutathione peroxidase 3
GSH: Glutathione
HGFAC: Hepatocyte growth factor activator
HRG: Histidine-rich glycoprotein
IGFBP1: IGF-binding protein 1
IGFBP2: IGF-binding protein 2
IGFBP3: IGF-binding protein 3
INNODIA: Innovative approaches to understanding and arresting type 1 diabetes
LMM: Linear mixed effects model
MASP2: MBL associated serine protease 2
MMTT: Mixed meal tolerance test
ND: Newly diagnosed with type 1 diabetes
PPARγ: Peroxisome proliferator-activated receptor γ
QC: Quality control
Se: Selenium
SERPING1: Plasma protease C1 inhibitor
SRM: Selected reaction monitoring
TGFBI: TGF-β-induced protein ig-h3
TTR: Transthyretin
UFM: Unaffected family member

## Members of the INNODIA and INNODIA HARVEST consortia

Mathieu C, Gillard P, Casteels K, Overbergh L (KU Leuven, Belgium), Dunger D, Wallace C, Evans M, Thankamony A, Hendriks E, Bruggraber S, Qureshi A, Marcovecchio L, Paediatrics laboratory staff (University of Cambridge, UK), Peakman M, Tree T (King’s College London, UK), Morgan N, Richardson S (University of Exeter, UK), Todd J, Wicker L (University of Oxford, UK), Mander A, Dayan C, Alhadj Ali M (Cardiff University, UK), Pieber T (Medical University of Graz, Austria), Eizirik D, Cnop M (Universite Libre de Bruxelles, Belgium), Brunak S (University of Copenhagen, Denmark), Pociot F, Johannesen J, Rossing P, Legido Quigley C (Herlev University Hospital, Region Hovedstaden, Denmark), Mallone R, Scharfmann R, Boitard C (Cochin Institute Paris, France), Knip M, Otonkoski T (University of Helsinki, Finland), Veijola R (University of Oulu, Finland), Lahesmaa R, Oresic M, Toppari J (University of Turku, Finland), Danne T (Children's and Youth Hospital Hannover, Germany), Ziegler AG, Achenbach P, Rodriguez-Calvo T (Helmholtz Zentrum Muenchen, Germany), Solimena M, Bonifacio E, Speier S (TU Dresden, Germany), Holl R (University of Ulm, Germany), Dotta F (University of Siena, Italy), Chiarelli F (University of Chieti, Italy), Marchetti P (University of Pisa, Italy), Bosi E (University Vita-Salute San Raffaele, Italy), Cianfarani S, Ciampalini P (Bambino Gesù Children's Hospital, Italy), de Beaufort C (Centre Hospitalier de Luxembourg, Luxemburg), Dahl-Jørgensen K, Skrivarhaug T, Joner G, Krogvold L (Oslo University Hospital, Norway), Jarosz-Chobot P (Medical University of Silesia, Poland), Battelino T (University of Ljubljana, Slovenia), Thorens B (University of Lausanne, Switzerland), Gotthardt M (Radboud University Medical Center, the Netherlands), Roep B, Nikolic T, Zaldumbide A (Leiden University Medical Center, the Netherlands), Lernmark A, Lundgren M (Lund University, Sweden), Costecalde G (Univercell-Biosolutions, France), Strube T, Schulte A, Nitsche A, (Sanofi, Germany), Peakman M, Vela J (Sanofi, United States), von Herrath M, Wesley J, (Novo Nordisk, Denmark), Napolitano-Rosen A (GlaxoSmithKline, UK), Thomas M, Schloot N (Eli Lilly, UK), Goldfine A, Waldron-Lynch F, Kompa J, Vedala A, Hartmann N, Nicolas G (Novartis Pharma AG, Switzerland), van Rampelbergh J, Bovy N (Imcyse SA, Belgium), Dutta S, Soderberg J, Ahmed S, Martin F, Latres E (JDRF, USA), Agiostratidou G, Koralova A (The Leona M. and Harry B. Helmsley Charitable Trust, USA).

## Associated clinical sites

Willemsen R (Barts Health NHS Trust, UK), Smith A (Northampton General Hospital NHS Trust, UK), Anand B (West Suffolk NHS FT, UK), Puthi V (North West Anglia NHS FT, UK), Zac-Varghese S (East & North Hertfordshire NHS Trust, UK), Datta V (Norfolk & Norwich University NHS FT, UK), Dias R (Birmingham Women’s and Children’s NHS FT, UK), Sundaram P (University Hospitals of Leicester NHS Trust, UK), Vaidya B (Royal Devon & Exeter NHS FT, UK), Patterson C (NHS Fife, UK), Owen K (Oxford University Hospitals NHS FT, UK), Dayan C (Cardiff & Vale University Health Board, UK), Piel B (Queen Elizabeth Hospital, King’s Lynn FT, UK), Heller S (Sheffield Teaching Hospitals NHS FT, UK), Randell T, Gazis T (Nottingham University Hospitals NHS Trust, UK), Bismuth Reismen E, Carel J-C (Hospital Robert Debre, France), Riveline J-P, Gautier J-F (Hospital Lariboisiere, France), Andreelli F (Hospital Lapitie-Salpetriere, France), Travert F (Hospital Bichat Claude Bernard, France), Cosson E (Hospital Jean-Verdier, France), Penfornis A, Petit C (Centre Hospitalier Sud-Francilien, France), Feve B (Hospital St Antoine, France), Lucidarme N (Hospital Jean-Verdier Pediatrie, France), Cosson E (Hospital Avicenne, France), Beressi J-P (Hospital Andre Mignot, France), Ajzenman C (Hospital Andre Mignot Pediatrie, France), Radu A (Hospital Europeen Georges-Pompidou, France), Greteau-Hamoumou S (Hospital Louis Mourier, France), Bibal C (Hospital Kremlin Bicetre, France), Meissner T (Universitatsklinikum der Heinrich-Heine-Univeritat Dusseldorf, Germany), Heidtmann B (Katholisches Kinderkrankenhaus Wilhelmstift, Germany), Toni S (AOU Meyer, Italy), Rami-Merhar B (Medical University of Vienna, Austria), Eeckhout B, Peene B, Vantongerloo N (Algemeen Ziekenhuis Geel Sint-Dimpna Geel, Belgium), Maes T, Gommers L (Imeldziekenhuis Bonheiden, Belgium).
