# Supplementary material for: Targeted serum proteomics of longitudinal samples from newly diagnosed youth with type 1 diabetes distinguishes markers of disease and C-peptide trajectory
Source: Diabetologia. 2023 Aug 4;66(11):1983–96. doi: 10.1007/s00125-023-05974-9 (PMC10542287; doi:10.1007/s00125-023-05974-9)
Supplement: Supplementary file 1 — (PDF 1.81 MB) [file 125_2023_5974_MOESM1_ESM.pdf]

SUPPLEMENTARY INFORMATION

Targeted serum proteomics of longitudinal samples from newly diagnosed youth with type 1 diabetes distinguishes markers of disease and C-peptide trajectory

Robert Moulder[1,2],Tommi Välikangas[1,2], M. Karoliina Hirvonen[1,2], Tomi Suomi[1,2], Caroline A. Brorsson[3], Niina Lietzén[1], Sylvaine F.A. Bruggraber[4], Lut Overbergh [5], David B. Dunger[4], Mark Peakman[6], Piotr J. Chmura[3], Soren Brunak[3], Anke M. Schulte[7], Chantal Mathieu[5], Mikael Knip[8,9,10], Laura L. Elo[1,2,11], Riitta Lahesmaa[1,2],

on behalf of the INNODIA consortium

[1]Turku Bioscience Centre, University of Turku and Åbo Akademi University, Turku, Finland
[2]InFLAMES Research Flagship Center, University of Turku, Turku, Finland
[3]Novo Nordisk Foundation Center for Protein Research, Faculty of Health and Medical Sciences, University of Copenhagen, Copenhagen, Denmark
[4]Department of Paediatrics, University of Cambridge, Cambridge, UK
[5]Katholieke Universiteit Leuven/Universitaire Ziekenhuizen, Leuven, Belgium
[6]Immunology & Inflammation Research Therapeutic Area, Sanofi, Boston, MA, USA
[7]Sanofi-Aventis Deutschland GmbH, Frankfurt, Germany
[8]Pediatric Research Center, University of Helsinki and Helsinki University Hospital, Helsinki, Finland;
[9]Research Program for Clinical and Molecular Metabolism, Faculty of Medicine, University of Helsinki, Helsinki, Finland;
[10]Tampere Center for Child Health Research, Tampere University Hospital, Tampere
[11]Institute of Biomedicine, University of Turku, Turku, Finland

David B. Dunger, who planned and designed this study, died on 20th July 2021 before publication of this work.

INNODIA - "Innovative approaches to understanding and arresting type 1 diabetes"

Contents

Protein Selection: Previous Type 1 Diabetes Proteomics studies 3
ESM Table 1: Proteins measured by Targeted Mass Spectrometry 4
a) Targets at a glance 4
b) Proteins selected from serum proteomics studies 5
c) References and classification of the other targeted proteins 7
ESM Table 2: Peptides Measured by targeted LC-MS/MS 8
INNODIA Project Overview 13
ESM Table 3: Samples compared in this study and the site of their collection 14
Additional technical Details 15
Sample preparation 15
Targeted LC-MS/MS 15
ESM Figure 1: Comparison of the targeted proteomics and fasting C-peptide/glucose data revealed several significant associations (FDR <0.05). 17

ESM Figure 2: Comparison of the targeted proteomics data from the age-matched ND and AAb-UFMs revealed significant differences for a series of peptides (FDR < 0.05). 19

ESM Figure 3: Correlation of the C-peptide/fasting glucose ratio and the mixed meal tolerance test (MMTT) area under the curve (AUC) values 22

ESM Figure 4: The distribution of Pearson correlation coefficients between the fasting C-peptide/glucose ratio (pmol/l for fasting C-peptide and mmol/l for fasting glucose) and the mixed meal tolerance test (MMTT) area under the curve (AUC) 23

References 24

Protein Selection: Previous Type 1 Diabetes Proteomics studies

Metz *et al.* used high-resolution capillary LC-MS/MS and intensity based quantification for the analysis of immuno-affinity depleted sera, reporting the first serum proteomics comparison of recently diagnosed T1D patients and matched controls (n=10 vs 10). They later followed this up in a second discovery study, using similar analytical methodology with pooled sera (n=10 vs 10) and a larger scale targeted mass spectrometry-based validation study (T1D=50, control =100, T2D = 50)[1,3]. From the panel of detected markers, they concluded that T1D caused dysregulation of innate immunity. From their validations they reported that peptides from platelet basic protein and C1 inhibitor (SEPRING1) achieved excellent discrimination between T1D and controls.

Zhi *et al.* used a spectral counting proteomics approach to quantify serum markers, comparing three pools of immuno-affinity depleted sera, each from 10 T1D patients with three similar controls pools[2]. From their panel of 21 candidate markers, immuno-assay validation was made for six targets in an extended cohort (1139 T1D and 849 Aab-). The latter targets included adiponectin, insulin-like growth factor binding protein 2, C-reactive protein, serum amyloid protein A, transforming growth factor beta induced and myeloperoxidase.

Oliveira *et al.*[7] used data-independent, label-free mass spectrometric analysis of immuno-affinity depleted sera to compare patients and control subjects (n=30 vs 30), reporting eight markers with overlap of some of the markers from the latter two studies.

T1D related proteomics studies have also included the analysis of sera from children who tested positive for T1D associated autoantibodies[6], temporal analysis of at-risk healthy controls and analysis of the changes leading to T1D onset[5]. In our previous investigations, we have used both isobaric labelling and label free methods to measured serum proteomes of children from a HLA-conferred T1D risk group to identify changes associated with maturation, the appearance of autoantibodies and diagnosis (n=19, vs n=19)[4,11].

In the study of islet autoantibody-positive children, von Toerne *et al.*[6] employed label free analysis of immuno-affinity depleted sera for discovery and targeted mass spectrometry-based validation. Here, they reported peptide signatures indicative islet autoimmunity could be found prior to the diagnosis of type 1 diabetes.

Liu and co-workers[5] used isobaric labelling of depleted sera to compare sera from children developing type 1 diabetes with age matched controls. They found statistically significant differences for 13 proteins, including catalase and superoxide dismutase, which were validated by ELISA. Taken together, they reported that oxidative stress related proteins were dysregulated before islet autoimmunity. Markers reported in the above studies and selected for measurement are indicated in the following tables.

ESM Table 1: Proteins measured by Targeted Mass Spectrometry

Targets were selected from a range of studies of type 1 diabetes and other serum/plasma proteomics investigations. For presentation, these are subdivided into general groups, with the control/reference proteins either in the control group or marked with †. The peptides measured are indicated in **ESM Table 2**

a) Targets at a glance. For quick reference, the selected proteins are tabulated with their gene name and group assignments. The controls proteins include albumin as basic indication of sample loading, fibrinogen (FGA) to rule out plasma samples and HBB as a marker of hemolysis.

**Apolipoprotein**	**Complement related**	**Acute Phase**	**Transmembrane receptor**	**Other**
APOA1	C1R	CRP	CD5L	ACAN
APOA4	C1S	SAA1	GP1BA†	ADIPOQ
APOB	C2	SAA2	IGHM	CLU
APOC1	C3	SAA4	LYVE1	COL1A1
APOC2	C4BPA	**Control**	PGLYRP2	CRISP3
APOC3 †	C5†	A1BG	SELP	DEFA3
APOC4	C6	ALB	**Peptidase**	FLNA1†
APOE	C8G	FGA	CNDP1	GSN
APOL1	C9	ACTB	F12	HRG
APOM	CFH	HBB	F2	IGJ
LPA	CFHR1	**Enzyme**	F9	LUM
**Serpin family**	CFHR4	BTD	HGFAC	MST1
SERPINA7	MASP2	CAT	HP	PFN1†
SERPIND1	MBL2	CP	KLKB1	PPBP
SERPINF2	**Insulin related**	CTBS	MMP2	PVR
SERPING1	IGF1	GAPDH†	MMP9	PZP
SERPINA6	IGF2	GPX3	**Transporter**	SCG3
	IGFALS	HSPA5	AFM	SPARC
	IGFBP1	KNG1	AZGP	SRGN
	IGFBP2	MPO	TF	TGFBI
	IGFBP3	PCYOX1	TTR	TREML1
		SOD1	GC†	VWF

b) Proteins selected from serum proteomics studies with references and group assignments [1–8]

Gene	Protein names	Accession	Literature Reference								Group
			1	2	3	**4**	**5**	6	7	8	
CRP	C-reactive protein	P02741		2							Acute Phase
SAA1	Serum amyloid A-1 protein	P0DJI8		2							Acute Phase
SAA2	Serum amyloid A-2 protein	P0DJI9		2							Acute Phase
SAA4	Serum amyloid A-4 protein	P35542		■		4					Acute Phase
APOA4	Apolipoprotein A-IV	P06727		2		4					Acute Phase
APOC2	Apolipoprotein C-II	P02655				4					Apolipoprotein
APOC4	Apolipoprotein C-IV	P55056				4					Apolipoprotein
APOE	Apolipoprotein E	P02649				4					Apolipoprotein
APOM	Apolipoprotein M	O95445						6			Apolipoprotein
APOC3†	Apolipoprotein C-III	P02656								8	Apolipoprotein
LPA	Apolipoprotein(a)	P08519				4					Apolipoprotein
C4BPA	C4b-binding protein alpha chain	P04003		2	3			6			Apolipoprotein
C3	Complement C3	P01024		2				6			Complement related
C1R	Complement C1r subcomponent	P00736			3						Complement related
C2	Complement C2	P06681			3						Complement related
C5†	Complement C5	P01031				4					Complement related
C6	Complement component C6	P13671			3						Complement related
C9	Complement component C9	P02748				4					Complement related
C8G	Complement component C8 gamma chain	P07360				4					Complement related
MASP2	Mannan-binding lectin serine protease 2	O00187				4					Complement related
MBL2	Mannose-binding protein C	P11226				4					Complement related
CFH	Complement factor H	P08603						6			Complement related
C1S	Complement C1s subcomponent	P09871							7		Complement related
HBB†	Hemoglobin subunit beta	P68871								8	Control
FGA†	Fibrinogen alpha chain	P02671								8	Control
ACTB†	Actin, cytoplasmic 1 (Beta-actin)	P60709								8	Control
HSPA5	Endoplasmic reticulum chaperone BiP	P11021	1								Control
BTD	Biotinidase	P43251			3			6			Enzyme
GPX3	Glutathione peroxidase 3	P22352			3						Enzyme
CAT	Catalase	P04040					5			8	Enzyme
SOD1	Superoxide dismutase [Cu-Zn]	P00441					5			8	Enzyme
CP	Ceruloplasmin	P00450						6			Enzyme
GAPDH†	Glyceraldehyde-3-phosphate dehydrogenase	P04406								8	Enzyme
IGFALS	Insulin-like growth factor-binding protein complex acid labile subunit	P35858	1			4					Enzyme
IGFBP2	Insulin-like growth factor-binding protein 2	P18065		■		4					Insulin related

PZP	Pregnancy zone protein	P20742	1						7		Insulin related
LUM	Lumican	P51884	1								Other
CLU	Clusterin	P10909	1								Other
ADIPOQ	Adiponectin	Q15848		2							Other
TGFBI	Transforming growth factor-beta-induced protein ig-h3	Q15582		2							Other
GSN	Gelsolin	P06396			3						Other
PPBP	Platelet basic protein	P02775			3						Other
TREML1	Trem-like transcript 1 protein	Q86YW5				4					Other
PVR	Poliovirus receptor	P15151				4					Other
MST1	Hepatocyte growth factor-like protein	P26927				4					Other
FLNA†	Filamin-A	P21333								8	Other
PFN1†	Profilin-1	P07737								8	Other
CNDP1	Beta-Ala-His dipeptidase	Q96KN2			3						Other
HGFAC	Hepatocyte growth factor activator	Q04756			3						Peptidase
KLKB1	Plasma kallikrein	P03952			3						Peptidase
F12	Coagulation factor XII	P00748				4					Peptidase
F9	Coagulation factor IX	P00740				4					Peptidase
F2	Prothrombin	P00734							7	8	Peptidase
SERPINA6	Corticosteroid-binding globulin	P08185	1		3						Peptidase
SERPINF2	Alpha-2-antiplasmin	P08697			3			6			Serpin family
SERPIND1	Heparin cofactor 2	P05546			3						Serpin family
SERPING1	Plasma protease C1 inhibitor	P05155			3						Serpin family
PGLYRP2	N-acetylmuramoyl-L-alanine amidase	Q96PD5			3						Serpin family
GP1BA†	Platelet glycoprotein Ib alpha chain	P07359								8	Transmembrane receptor
TF	Serotransferrin	P02787	1								Transmembrane receptor
AZGP1	Zinc-alpha-2-glycoprotein	P25311	1								Transporter
TTR	Transthyretin	P02766			3			6			Transporter
AFM	Afamin	P43652				4					Transporter
GC†	Vitamin D-binding protein	P02774								8	Transporter

[1]Metz TO, et al. J Proteome Res. 2008; [2] Zhi W, et al. Mol Cell Proteomics. 2011; [3] Zhang Q, et al. J Exp Med. 2013; [4]Moulder R, et al. Diabetes. 2015; [5]Liu CW, et al. J Proteomics. 2018; [6]von Toerne C, et al.. Diabetologia. 2016; [7]Oliveira, et al. Diabetes Metab Syndr Obes. 2018; [8]Geyer PE, et al. EMBO Mol Med. 2019. † = selected as control/marker protein

c) References and classification of the other targeted proteins are as follows

**Gene**	**Protein names**	**Accession**	**Ref**	**Group**
APOC1	Apolipoprotein C-I	P02654	9	Apolipoprotein
APOL1	Apolipoprotein L1	O14791	10	Apolipoprotein
APOA1	Apolipoprotein A-I	P02647	11	Apolipoprotein
APOB	Apolipoprotein B-100 [Cleaved into: Apo B-48]	P04114	12	Apolipoprotein
CFHR1	Complement factor H-related protein 1	Q03591	ƚ	Complement related
CFHR4	Complement factor H-related protein 4	Q92496	ƚ	Complement related
ALB	Albumin	P02768	†	Control
A1BG	Alpha-1B-glycoprotein	P04217	†	Control
KNG1	Kininogen-1	P01042	†	Control
PCYOX1	Prenylcysteine oxidase 1	Q9UHG3	ƚ	Enzyme
CTBS	Di-N-acetylchitobiase	Q01459	ƚ	Enzyme
MPO	Myeloperoxidase	P05164	ƚ	Enzyme
IGFBP1	Insulin-like growth factor-binding protein 1	P08833	13	Insulin related
IGF1	Insulin-like growth factor I	P05019	14	Insulin related
IGFBP3	Insulin-like growth factor-binding protein 3	P17936	14	Insulin related
IGF2	Insulin-like growth factor II	P01344	ƚ	Insulin related
DEFA3	Neutrophil defensin 3	P59666	11	Other
IGJ	Immunoglobulin J chain	P01591	11	Other
ACAN	Aggrecan core protein	P16112	ƚ	Other
COL1A1	Collagen alpha-1(I) chain	P02452	ƚ	Other
SRGN	Serglycin	P10124	ƚ	Other
CRISP3	Cysteine-rich secretory protein 3	P54108	ƚ	Other
HRG	Histidine-rich glycoprotein	P04196	ƚ	Other
SCG3	Secretogranin-3	Q8WXD2	ƚ	Other
SPARC	SPARC (Secreted protein acidic and rich in cysteine)	P09486	ƚ	Other
VWF	von Willebrand factor	P04275	15	Other
MMP2	72 kDa type IV collagenase (Matrix metalloproteinase-2)	P08253	16	Peptidase
HP	Haptoglobin (Zonulin)	P00738	17	Peptidase
MMP9	Matrix metaloproteinase-9	P14780	18	Peptidase
SERPINA7	Thyroxine-binding globulin (Serpin A7)	P05543	19	Serpin family
SELP	P-selectin	P16109	15	Transmembrane receptor
CD5L	CD5 antigen-like	O43866	ƚ	transmembrane receptor
LYVE1	Lymphatic vessel endothelial hyaluronic acid receptor 1	Q9Y5Y7	ƚ	transmembrane receptor
IGHM	Immunoglobulin heavy constant mu	P01871	ƚ	transmembrane receptor

[9]Albrethsen J, et al. *Proteomics Clin Appl*. 2009; [10]Duchateau PN, et al. *J Lipid Res*. 2000; [11]Lietzen N, et al. *Sci Rep*. 2018; [12]Malmström R, *Diabetes*. 1998; [13]Bereket A, et al. *J Clin Endocrinol Metab*. 1995; [14]Peet A, *Eur J Endocrinol*. 2015; [15]Jilma B, *Thromb Haemost*. 1996; [16]Thrailkill KM, et al., *Diabetes Care*. 2007; [17]Sapone A, et al. *Diabetes*. 2006; [18]Jacqueminet S, et al. *Clin Chim Acta*. 2006; [19]Connors MH, et al. *Diabetes Care*. 1996. ƚ = From INNODIA researchers, † = selected as control/marker protein

SUPPLEMENTARY INFORMATION

ESM Table 2: Peptides Measured by targeted LC-MS/MS

Peptides	Protein Accession	Protein name	Gene
AGFAGDDAPR	C9JTX5	Actin, cytoplasmic 1 (Fragment)	ACTB
AVFPSIVGR	C9JTX5	Actin, cytoplasmic 1 (Fragment)	ACTB
LASPGFPGEYANDQER	O00187	Mannan-binding lectin serine protease 2	MASP2
VLATLCGQESTDTER	O00187	Mannan-binding lectin serine protease 2	MASP2
VTEPISAESGEQVER	O14791	Apolipoprotein L1	APOL1
EATLQDCPSGPWGK	O43866	CD5 antigen-like	CD5L
IWLDNVR	O43866	CD5 antigen-like	CD5L
AFLLTPR	O95445	Apolipoprotein M	APOM
SLTSCLDSK	O95445	Apolipoprotein M	APOM
TLVVHEK	P00441	Superoxide dismutase [Cu-Zn]	SOD1
DDEEFIESNK	P00450	Ceruloplasmin	CP
DIASGLIGPLIICK	P00450	Ceruloplasmin	CP
TATSEYQTFFNPR	P00734	Prothrombin	F2
GLTLHLK	P00736	Complement C1r subcomponent	C1R
HYEGSTVPEK	P00738	Haptoglobin	HP
VVLHPNYSQVDIGLIK	P00738	Haptoglobin	HP
NPANPVQ	H0Y300	Haptoglobin	HP
NPANPVQR	H0Y300	Haptoglobin	HP
FGSGYVSGWGR	P00740	Coagulation factor IX	F9
SALVLQYLR	P00740	Coagulation factor IX	F9
NGPLSCGQR	P00748	Coagulation factor XII	F12
AAVYHHFISDGVR	P01024	Complement C3	C3
ENSQYQPIK	P01031	Complement C5	C5
TDAPDLPEENQAR	P01031	Complement C5	C5
QVVAGLNFR	P01042	Kininogen-1	KNG1
TVGSDTFYSFK	P01042	Kininogen-1	KNG1
GIVEECCFR	P01344	Insulin-like growth factor II	IGF2
FVYHLSDLCK	P01591	Insulin-like growth factor II	IGJ
SSEDPNEDIVER	P01591	Immunoglobulin J chain	IGJ
QVGSGVTTDQVQAEAK	P01871	Immunoglobulin heavy constant mu	IGHM
YAATSQVLLPSK	P01871	Immunoglobulin heavy constant mu	IGHM
ICVCDNGK	P02452	Collagen alpha-1(I) chain	COL1A1
VLCDDVICDETK	P02452	Collagen alpha-1(I) chain	COL1A1
DYVSQFEGSALGK	P02647	Apolipoprotein A-I	APOA1
THLAPYSDELR	P02647	Apolipoprotein A-I	APOA1
LEEQAQQIR	P02649	Apolipoprotein E	APOE
LGPLVEQGR	P02649	Apolipoprotein E	APOE
EFGNTLEDK	P02654	Apolipoprotein C-I	APOC1
EWFSETFQK	P02654	Apolipoprotein C-I	APOC1
TAAQNLYEK	P02655	Apolipoprotein C-II	APOC2
TYLPAVDEK	P02655	Apolipoprotein C-II	APOC2
DYWSTVK	P02656	Apolipoprotein C-III	APOC3

GWVTDGFSSLK	P02656	Apolipoprotein C-III	APOC3
ESSSHHPGIAEFPSR	P02671	Fibrinogen alpha chain	FGA
GSESGIFTNTK	P02671	Fibrinogen alpha chain	FGA
AFVFPK	P02741	C-reactive protein	CRP
ESDTSYVSLK	P02741	C-reactive protein	CRP
GYSIFSYATK	P02741	C-reactive protein	CRP
ALPTTYEK	P02748	Complement component C9	C9
FTPTETNK	P02748	Complement component C9	C9
LSPIYNLVPVK	P02748	Complement component C9	C9
AADDTWEPFASGK	P02766	Transthyretin	TTR
TSESGELHGLTTEEEFVEGIYK	P02766	Transthyretin	TTR
YLYEIAR	P02768	Albumin	ALB
HLSLLTTLSNR	P02774	Vitamin D-binding protein	GC
VLEPTLK	P02774	Vitamin D-binding protein	GC
NIQSLEVIGK	P02775	Platelet basic protein	PPBP
DGAGDVAFVK	P02787	Serotransferrin	TF
EDPQTFYYAVAVVK	P02787	Serotransferrin	TF
DSVTGTLPK	P03952	Plasma kallikrein	KLKB1
IAYGTQGSSGYSLR	P03952	Plasma kallikrein	KLKB1
VSEGNHDIALIK	P03952	Plasma kallikrein	KLKB1
EEIIYECDK	P04003	C4b-binding protein alpha chain	C4BPA
GSSVIHCDADSK	P04003	C4b-binding protein alpha chain	C4BPA
YTCLPGYVR	P04003	C4b-binding protein alpha chain	C4BPA
ADVLTTGAGNPVGDK	P04040	Catalase	CAT
FSTVAGESGSADTVR	P04040	Catalase	CAT
ENFAGEATLQR	P04114	Apolipoprotein B-100	APOB
EVGTVLSQVYSK	P04114	Apolipoprotein B-100	APOB
ITENDIQIALDDAK	P04114	Apolipoprotein B-100	APOB
ADLFYDVEALDLESPK	P04196	Histidine-rich glycoprotein	HRG
DGYLFQLLR	P04196	Histidine-rich glycoprotein	HRG
ATWSGAVLAGR	P04217	Alpha-1B-glycoprotein	A1BG
SGLSTGWTQLSK	P04217	Alpha-1B-glycoprotein	A1BG
ILAGPAGDSNVVK	P04275	von Willebrand factor	VWF
VTVFPIGIGDR	P04275	von Willebrand factor	VWF
GALQNIIPASTGAAK	P04406	Glyceraldehyde-3-phosphate dehydrogenase	GAPDH
LVINGNPITIFQER	P04406	Glyceraldehyde-3-phosphate dehydrogenase	GAPDH
APQTGIVDECCFR	P05019	Insulin-like growth factor I	IGF1
GFYFNKPTGYGSSSR	P05019	Insulin-like growth factor I	IGF1
LLDSLPSDTR	P05155	Plasma protease C1 inhibitor	SERPING1
TLYSSSPR	P05155	Plasma protease C1 inhibitor	SERPING1
IANVFTNAFR	P05164	Myeloperoxidase	MPO
NALALFVLPK	P05543	Thyroxine-binding globulin	SERPINA7
TLEAQLTPR	P05546	Heparin cofactor 2	SERPIND1
AGALNSNDAFVLK	P06396	Gelsolin	GSN
TPSAAYLWVGTGASEAEK	P06396	Gelsolin	GSN

AVISPGFDVFAK	P06681	Complement C2	C2
HAFILQDTK	P06681	Complement C2	C2
ISASAEELR	P06727	Apolipoprotein A-IV	APOA4
LAPLAEDVR	P06727	Apolipoprotein A-IV	APOA4
LTQLNLDR	P07359	Platelet glycoprotein Ib alpha chain	GP1BA
LTSLPLGALR	P07359	Platelet glycoprotein Ib alpha chain	GP1BA
SLPVSDSVLSGFEQR	P07360	Complement component C8 gamma chain	C8G
VQEAHLTEDQIFYFPK	P07360	Complement component C8 gamma chain	C8G
STGGAPTFNVTVTK	P07737	Profilin-1	PFN1
HLVALSPK	P08185	Corticosteroid-binding globulin	SERPINA6
ITQDAQLK	P08185	Corticosteroid-binding globulin	SERPINA6
AFQVWSDVTPLR	P08253	72 kDa type IV collagenase	MMP2
GTYSTTVTGR	P08519	Apolipoprotein	LPA
NPDAVAAPYCYTR	P08519	Apolipoprotein	LPA
CTSTGWIPAPR	P08603	Complement factor H	CFH
IDVHLVPDR	P08603	Complement factor H	CFH
DFLQSLK	P08697	Alpha-2-antiplasmin	SERPINF2
LFGPDLK	P08697	Alpha-2-antiplasmin	SERPINF2
AQETSGEEISK	P08833	Insulin-like growth factor-binding protein 1	IGFBP1
LHLDYIGPCK	P09486	SPARC	SPARC
TFDSSCHFFATK	P09486	SPARC	SPARC
EDTPNSVWEPAK	P09871	Complement C1s subcomponent	C1S
FFGHGAEDSLADQAANEWGR	P0DJI8	Serum amyloid A-1 protein	SAA1
GPGGVWAAEAISDAR	P0DJI8	Serum amyloid A-1 protein	SAA1
GAEDSLADQAANK	P0DJI9	Serum amyloid A-2 protein	SAA2
GPGGAWAAEVISNAR	P0DJI9	Serum amyloid A-2 protein	SAA2
CNPDSNSANCLEEK	P10124	Serglycin	SRGN
LRTDLFPK	P10124	Serglycin	SRGN
ASSIIDELFQDR	P10909	Clusterin	CLU
ITPSYVAFTPEGER	P11021	Endoplasmic reticulum chaperone BiP	HSPA5
NELESYAYSLK	P11021	Endoplasmic reticulum chaperone BiP	HSPA5
FQASVATPR	P11226	Mannose-binding protein C	MBL2
GFVVAGPSR	P13671	Complement component C6	C6
IGESIELTCPK	P13671	Complement component C6	C6
SLGPALLLLQK	P14780	Matrix metalloproteinase-9	MMP9
SVDIWLR	P15151	Poliovirus receptor	PVR
VLAKPQNTAEVQK	P15151	Poliovirus receptor	PVR
FECQPGYR	P16109	P-selectin	SELP
LEGPNNVECTTSGR	P16109	P-selectin	SELP
LEGEVFFATR	P16112	Aggrecan core protein	ACAN
ETEYGPCR	P17936	Insulin-like growth factor-binding protein 3	IGFBP3
FLNVLSPR	P17936	Insulin-like growth factor-binding protein 3	IGFBP3
SAGSVESPSVSSTHR	P17936	Insulin-like growth factor-binding protein 3	IGFBP3
LEGEACGVYTPR	P18065	Insulin-like growth factor-binding protein 2	IGFBP2

LIQGAPTIR	P18065	Insulin-like growth factor-binding protein 2	IGFBP2
GSFALSFPVESDVAPIAR	P20742	Pregnancy zone protein	PZP
ISEITNIVSK	P20742	Pregnancy zone protein	PZP
AGQSAAGAAPGGGVDTR	P21333	Filamin-A	FLNA
VEPGLGADNSVVR	P21333	Filamin-A	FLNA
WGDEHIPGSPYR	P21333	Filamin-A	FLNA
FLVGPDGIPIMR	P22352	Glutathione peroxidase 3	GPX3
AGEVQEPELR	P25311	Zinc-alpha-2-glycoprotein	AZGP1
EIPAWVPFDPAAQITK	P25311	Zinc-alpha-2-glycoprotein	AZGP1
YSLTYIYTGLSK	P25311	Zinc-alpha-2-glycoprotein	AZGP1
SPLNDFQVLR	P26927	Hepatocyte growth factor-like protein	MST1
TPFDYCALR	P26927	Hepatocyte growth factor-like protein	MST1
GPGGVWAAK	P35542	Serum amyloid A-4 protein	SAA4
YLYAR	P35542	Serum amyloid A-4 protein	SAA4
LAELPADALGPLQR	P35858	Insulin-like growth factor-binding protein complex acid labile subunit	IGFALS
LEALPNSLLAPLGR	P35858	Insulin-like growth factor-binding protein complex acid labile subunit	IGFALS
LEYLLLSR	P35858	Insulin-like growth factor-binding protein complex acid labile subunit	IGFALS
LSSGLVTAALYGR	P43251	Biotinidase	BTD
WNPCLEPHR	P43251	Biotinidase	BTD
AESPEVCFNEESPK	P43652	Afamin	AFM
DADPDTFFAK	P43652	Afamin	AFM
GQCIINSNK	P43652	Afamin	AFM
FNALQYLR	P51884	Lumican	LUM
ILGPLSYSK	P51884	Lumican	LUM
AVSPPAR	P54108	Cysteine-rich secretory protein 3	CRISP3
YEDLYSNCK	P54108	Cysteine-rich secretory protein 3	CRISP3
AWFLESK	P55056	Apolipoprotein C-IV	APOC4
ELLETVVNR	P55056	Apolipoprotein C-IV	APOC4
WSLVR	P55056	Apolipoprotein C-IV	APOC4
IPACIAGER	P59666	Neutrophil defensin 3	DEFA3
YGTCIYQGR	P59666	Neutrophil defensin 3	DEFA3
SAVTALWGK	P68871	Hemoglobin subunit beta	HBB
VNVDEVGGEALGR	P68871	Hemoglobin subunit beta	HBB
ATYIQNYR	Q01459	Di-N-acetylchitobiase	CTBS
STDTSCVNPPTVQNAHILSR	Q03591	Complement factor H-related protein 1	CFHR1
TGESAEFVCK	Q03591	Complement factor H-related protein 1	CFHR1
LEACESLTR	Q04756	Hepatocyte growth factor activator	HGFAC
VANYVDWINDR	Q04756	Hepatocyte growth factor activator	HGFAC
LTLLAPLNSVFK	Q15582	Transforming growth factor-beta-induced protein ig-h3	TGFBI
GFPGIQGR	Q15848	Adiponectin	ADIPOQ
IFYNQQNHYDGSTGK	Q15848	Adiponectin	ADIPOQ
FLPEGCQPLVSSAVDR	Q86YW5	Trem-like transcript 1 protein	TREML1

VSLNILPPEEEEEETHK	Q86YW5	Trem-like transcript 1 protein	TREML1
TLIDFVK	Q8WXD2	Secretogranin-3	SCG3
VEYQCQSYYELQGSK	Q92496	Complement factor H-related protein 4	CFHR4
VYLPWSR	Q92496	Complement factor H-related protein 4	CFHR4
AIHLDLEEYR	Q96KN2	Beta-Ala-His dipeptidase	CNDP1
ALEQDLPVNIK	Q96KN2	Beta-Ala-His dipeptidase	CNDP1
AGLLRPDYALLGHR	Q96PD5	N-acetylmuramoyl-L-alanine amidase	PGLYRP2
TFTLLDPK	Q96PD5	N-acetylmuramoyl-L-alanine amidase	PGLYRP2
IFSQETLTK	Q9UHG3	Prenylcysteine oxidase 1	PCYOX1
SDFYDIVLVATPLNR	Q9UHG3	Prenylcysteine oxidase 1	PCYOX1
LLGLSLAGK	Q9Y5Y7	Lymphatic vessel endothelial hyaluronic acid receptor 1	LYVE1
LGGNETQVR	MSRT1	Isotopically labelled retention time std.	
ADEGISFR	MSRT1	Isotopically labelled retention time std.	
AEFAEVSK	MSRT1	Isotopically labelled retention time std.	
AVQQPDGLAVLGIFLK	MSRT1	Isotopically labelled retention time std.	
DISLSDYK	MSRT1	Isotopically labelled retention time std.	
DQGGELLSLR	MSRT1	Isotopically labelled retention time std.	
GLFIIDDK	MSRT1	Isotopically labelled retention time std.	
LVNEVTEFAK	MSRT1	Isotopically labelled retention time std.	
SGFSSVSVSR	MSRT1	Isotopically labelled retention time std.	
TDELFQIEGLKEELAYLR	MSRT1	Isotopically labelled retention time std.	
YWGVASFLQK	MSRT1	Isotopically labelled retention time std.	

INNODIA Project Overview:

https://www.innodia.eu/

The INNODIA project (Innovative approaches to understanding and arresting type 1 diabetes) was established in 2015 as a pan-European consortium with the principle aim to fight T1D. Participating clinical centres in thirteen countries have monitored newly diagnosed individuals and unaffected family members, collecting biological samples to define the natural history of the disease. Subsequent actions in the study have included the launch of clinical trials with immunosuppressive drugs to prolong beta cell function (https://www.innodia.eu/harvest/).

As an early project milestone, detailed analysis of blood samples collected from first 100 newly diagnosed (ND) individuals have been made. These multi-omics analyses, targeting microRNA, mRNA, metabolites, lipids, proteins, and immune cell populations, together with data on C -peptide levels, fasting glucose and mixed meal tolerance tests, present a baseline for reference in the newly diagnosed, and are to be described elsewhere.

The samples analysed in this proteomics study were collected between 2018 and 2019 from the first 100 subject newly diagnosed with type 1 diabetes. With this consecutive recruitment approach, subjects between the ages of one to 45 years were included on the basis of an even gender distribution, biosample availability and positivity for at least one diabetes-related autoantibody (GADA, IA-2A, ZnT8A). To reduce the influence of age in these targeted proteomics comparisons, only individuals diagnosed up to the age of 18 were considered, together with age and sex matched unaffected members of type 1 diabetes families. The data from the newly diagnosed were compared with C-peptide and fasting glucose measurements (used as a ratio of C-peptide/glucose). C-peptide data was collected up until 2020. The samples from the newly diagnosed included blood/sera drawn within 6 weeks of diagnosis, then 3 months, 6 months and up to one year afterwards.

ESM Table 3: Samples compared in this study and the site of their collection.

**Center**	ND (n)	ND age range (medium)	AAb- UFM (n)	UFM age range (medium)
Luxemborg, LUX	2	6-16 (11)	4	2-17 (16)
Herlev, DK	17	4-18 (12)	15	5-16 (11)
Hannover, DE	1	2-2 (2)	14	2-15 (11)
Leuven, BE	1	17-17 (17)	11	5-17 (16)
Graz, AT	1	13-13 (13)	20	3-13 (10)
Katowice, PL	3	9-12 (9)	3	5-9 (5)
Cambridge, UK	6	7-18 (12)	20	7-17 (11)
Helsinki, FI	28	1-16 (7)	44	1-17 (6.5)
London, UK	3	8-11 (11)	0	NA
Lister, UK	0	NA	3	4-9 (9)
Norwich, UK	2	3-15 (9)	5	4-12 (7)
Peterborough, UK	0	NA	2	2-8 (5)
Northampton, UK	0	NA	2	12-15 (13.5)
Birmingham, UK	0	NA	1	12-12 (12)
Bruxelles, BE	2	14-17 (15.5)	5	6-17 (14)
Ljubljana, SL	18	4-13 (11)	9	7-17 (8)
Siena, ITA	1	18-18 (18)	0	NA
Olun, FI	1	3-3 (3)	35	1-17 (9)
Ulm, DE	0	NA	1	4-4 (4)
**Sex Distribution**				
Female (total)	41		91	
Male (total)	45		103	

Additional technical Details

Sample preparation

The serum samples were prepared in eight batches in eight 96-well plates. Three distinct quality control (QC) serum samples within each batch. The plates were designed to include newly diagnosed individuals and selected sex and age matched unaffected family members, and randomised for the order of sample analysis.

Serum (4 µl) was diluted with 8M urea in Tris-HCl (100 µl x 50 mM), reduced in 10 mM DTT (1-hour, 37 ˚C), and alkylated with 10 mM IAA in darkness (30 min, room temperature). Half the sample (55 µl) was aliquoted and diluted with Tris-HCl (400 µl x 50 mM), digested with trypsin (4.3 µg) with an estimated 1:30 ratio of trypsin:protein at 37 ^{0}C overnight, then quenched with 10% TFA. The digests were desalted using 96 well SepPak 100 mg reversed phase SPE plates (SepPak C18, Waters, SKU: 186002321), as previously described[20,21]

The desalted sample were dried using a vacuum centrifuge (SAVANT SPD1010, Thermo Scientific), reconstituted in MilliQ water containing 2% formic acid, 2% acetonitrile, and the peptide recovery estimated by absorbance at 280 nm using a NanoDrop-1000 UV spectrophotometer (Thermo Scientific). The samples were diluted to 50 ng/ul, including a spiked solution of heavy isotope-labelled synthetic peptides analogues spiked (~10fmol/µl, typically two peptides per protein, PEPotec, Thermo Fischer Scientific) and retention time standards (MSRT1, Sigma).

Targeted LC-MS/MS

Skyline software[22] was used to develop the retention time-scheduled data acquisition method and subsequently check the peak detection and integration

Retention time scheduled selected reaction monitoring (SRM) mass spectrometry (MS) was used as previously described[20]. In brief, a TSQ Vantage Triple Quadrupole Mass Spectrometer (Thermo Scientific) was coupled with an Easy-nLC 1000 liquid chromatograph (Thermo Scientific). A 20 mm x 100 µm i.d loading-column and a 150 mm x 75 µm i.d. analytical column, both packed with 5 µm Reprosil C18-bonded silica (Dr Maisch GmbH) were used.

A separation gradient was used from 5 to 21% B in 19 min, then to 36% B in 14 min, to 100% B in 4 minutes, with 8 minutes isochratic period at 100% B and flow rate of 300 nl/min (Solvent A: 0.1% formic acid in Milli-Q® H_2O and Solvent B: 80% acetonitrile, 0.1% formic acid in Milli-Q® H_2O).

The estimated peptide amount analysed was 250 ng, based on 5 µl x 0.05 µg/ µl.

The age matched samples were divided such that they were prepared in the same 96-well plates, and then analysed in the same batches, with the QCs samples included periodically in each batch.

SUPPLEMENTARY INFORMATION

There were 98 proteins targets, including 85 with type 1 diabetes associations and 13 selected for reference or otherwise, with 160 and 35 associated peptides respectively. Typically, there were two peptides per protein with 3-8 transitions per peptide.
The selected peptides were chosen on the basis of consistent detection in serum analysis from earlier reports, including our own data, and are indicated in ESM Table 2. Comparison with measurements from a previous study of serum glycation revealed that glycated forms of these peptides had not been observed.

Both the heavy and light peptides were measured throughout. The elution profiles were compared with the heavy peptides and corrected where appropriate. Transitions that could not be consistently corrected to ensure profiles matching the synthetic peptide were removed.

ESM Figure 1: Comparison of the targeted proteomics and fasting C-peptide/glucose data revealed several significant associations (FDR <0.05). The data is represented as locally estimated scatterplot smoothing (LOESS) curves for the normalized peptide abundances (red) and fasting C-peptide/Glucose (grey) from all the ND subjects relative to sampling time after disease onset. The LOESS curves are represented by the solid lines and their 95% confidence intervals by the dashed lines. The peptide expression data and the fasting C-peptide / glucose ratio data were adjusted for the potential confounding factors gender, height, standardized BMI (BMI_SDS), study centre and individual variation. Both the fasting C-peptide / glucose ratio (log_e transformed, pmol/l for fasting C-peptide and mmol/l for fasting glucose) and the peptide expression (normalized log_2 transformed intensity) data are scaled (z-score standardized) within each feature and the scaled peptide expression values are further offset by -2 for visualization of the respective changes of both variables during the first year after diagnosis in the same plot. Individual data points are shown as grey for the fasting C-peptide / glucose ratio and red for the peptide expression values to display the heterogeneity of the data. A small amount of random variation is added to the time value of each measurement for better visualization. Similarly, the scale of the Y-axis is limited between the 5th and 95th percentiles for the exclusion of extreme values for better visual presentation of the data.

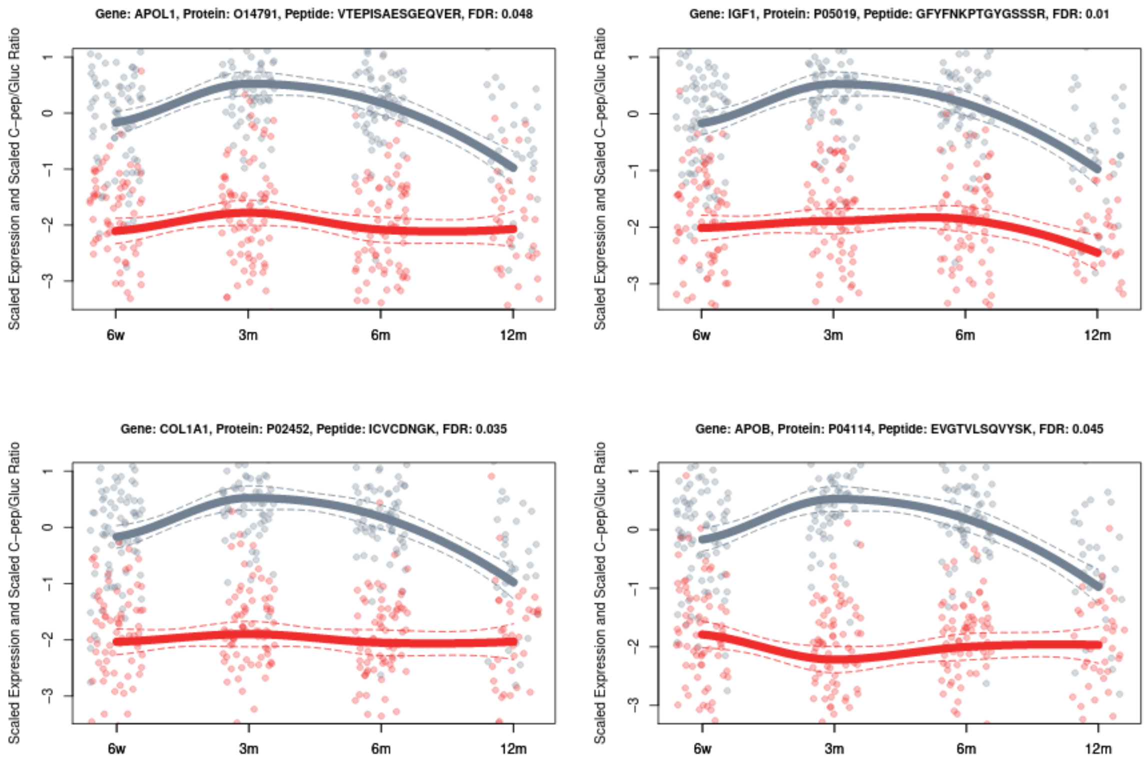

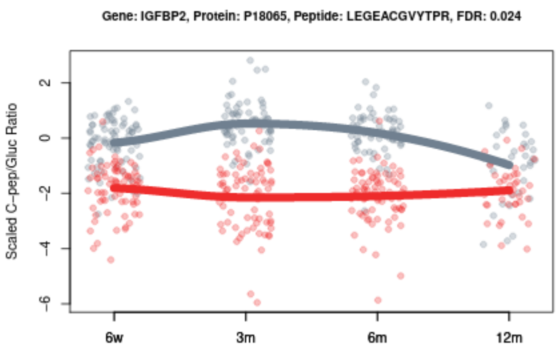
Gene: IGFBP2, Protein: P18065, Peptide: LEGEACGVYTPR, FDR: 0.024
Scaled C−pep/Gluc Ratio
6w
3m
6m
12m

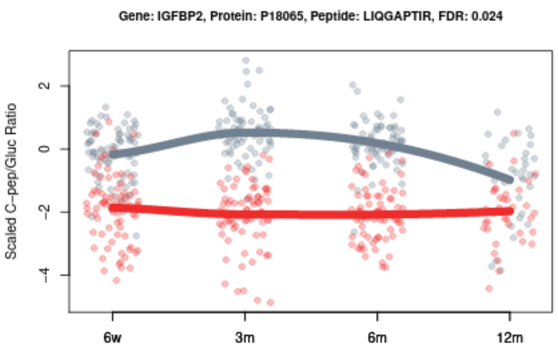
Gene: IGFBP2, Protein: P18065, Peptide: LIQGAPTIR, FDR: 0.024
Scaled C−pep/Gluc Ratio
6w
3m
6m
12m

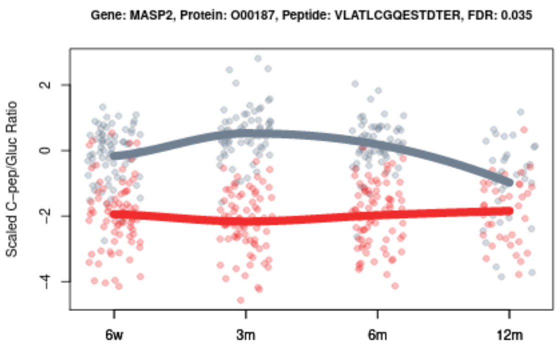
Gene: MASP2, Protein: O00187, Peptide: VLATLCGQESTDTER, FDR: 0.035
Scaled C−pep/Gluc Ratio
6w
3m
6m
12m

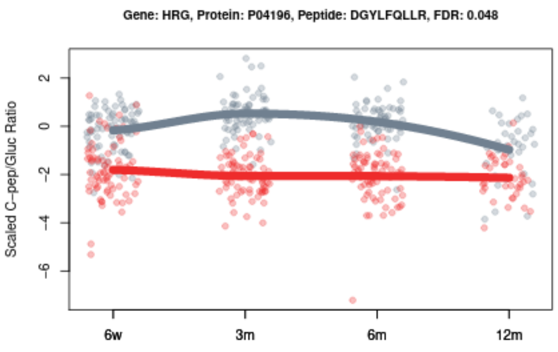
Gene: HRG, Protein: P04196, Peptide: DGYLFQLLR, FDR: 0.048
Scaled C−pep/Gluc Ratio
6w
3m
6m
12m

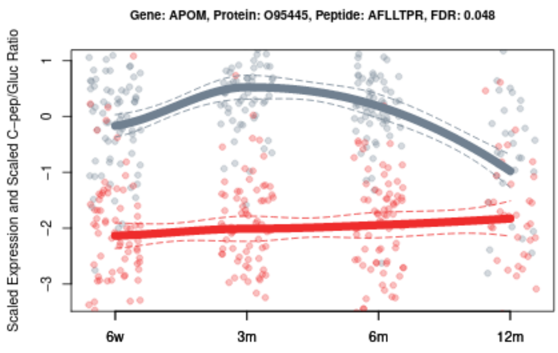
Gene: APOM, Protein: O95445, Peptide: AFLLTPR, FDR: 0.048
Scaled Expression and Scaled C−pep/Gluc Ratio
6w
3m
6m
12m

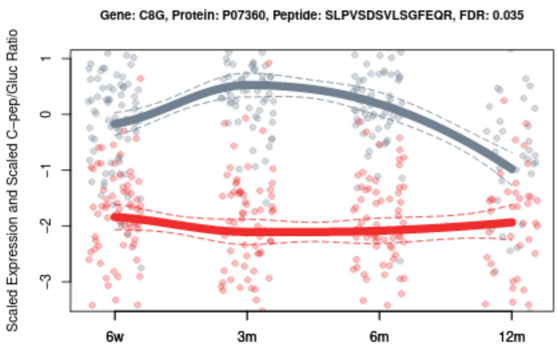
Gene: C8G, Protein: P07360, Peptide: SLPVSDSVLSGFEQR, FDR: 0.035
Scaled Expression and Scaled C−pep/Gluc Ratio
6w
3m
6m
12m

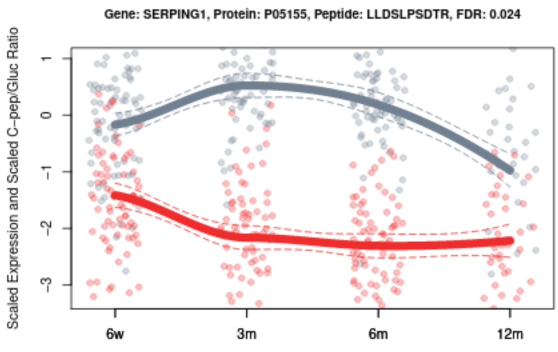
Gene: SERPING1, Protein: P05155, Peptide: LLDSLPSDTR, FDR: 0.024
Scaled Expression and Scaled C−pep/Gluc Ratio
6w
3m
6m
12m

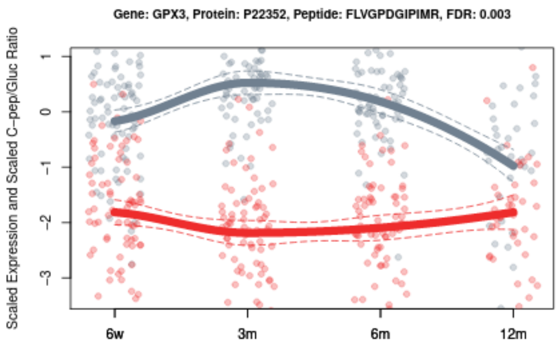
Gene: GPX3, Protein: P22352, Peptide: FLVGPDGIPIMR, FDR: 0.003
Scaled Expression and Scaled C−pep/Gluc Ratio
6w
3m
6m
12m

ESM Figure 2: Comparison of the targeted proteomics data from the age-matched ND and AAb- UFMs revealed significant differences for a series of peptides (FDR < 0.05). The data is represented as the locally estimated scatterplot smoothing (LOESS) curves for the peptide abundances (normalized log_2 transformed intensity) relative to age and grouped according to status, i.e. ND subjects (red) and AAb- UFMs (blue). The LOESS curves are represented by the solid lines and their 95% confidence intervals by the dashed lines. Individual data points are shown as red for the ND subjects and blue for the AAb- UFMs to display the heterogeneity of the data. A small amount of random variation is added to the age value of each measurement for better visualization. Similarly, the scale of the Y-axis is limited between the 5th and 95th percentiles for the exclusion of extreme values for better visual presentation of the data.

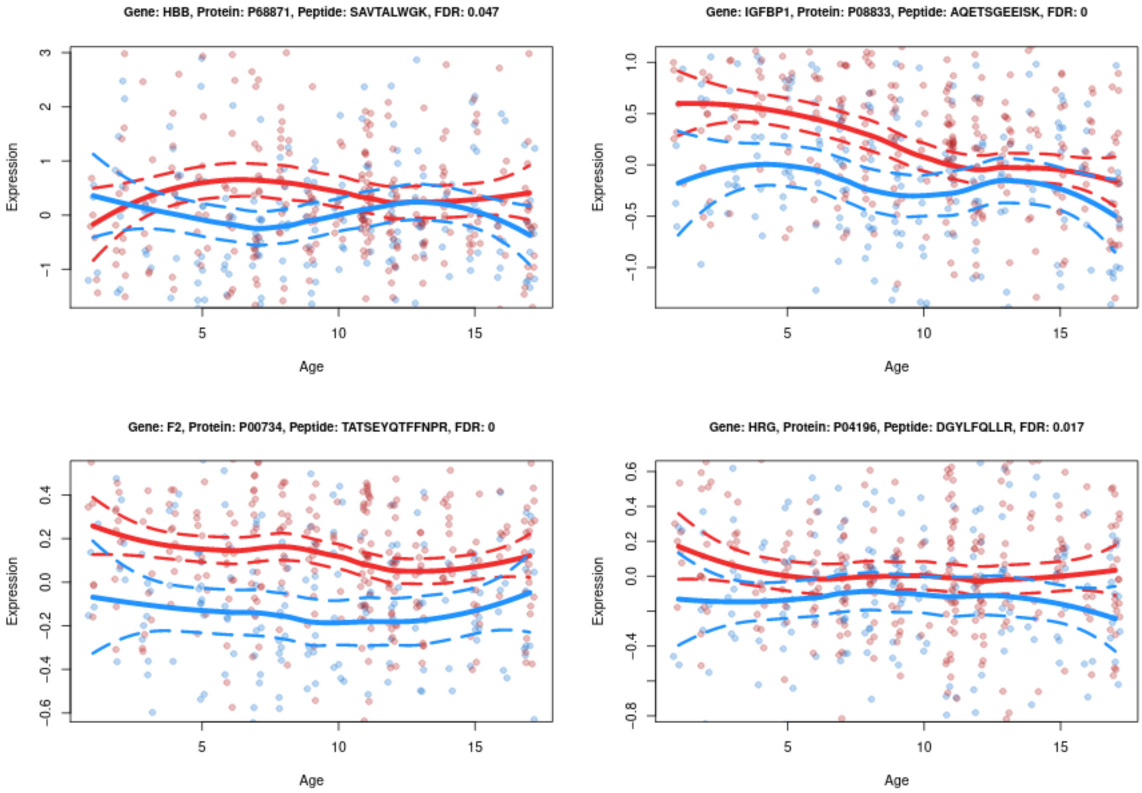

SUPPLEMENTARY INFORMATION

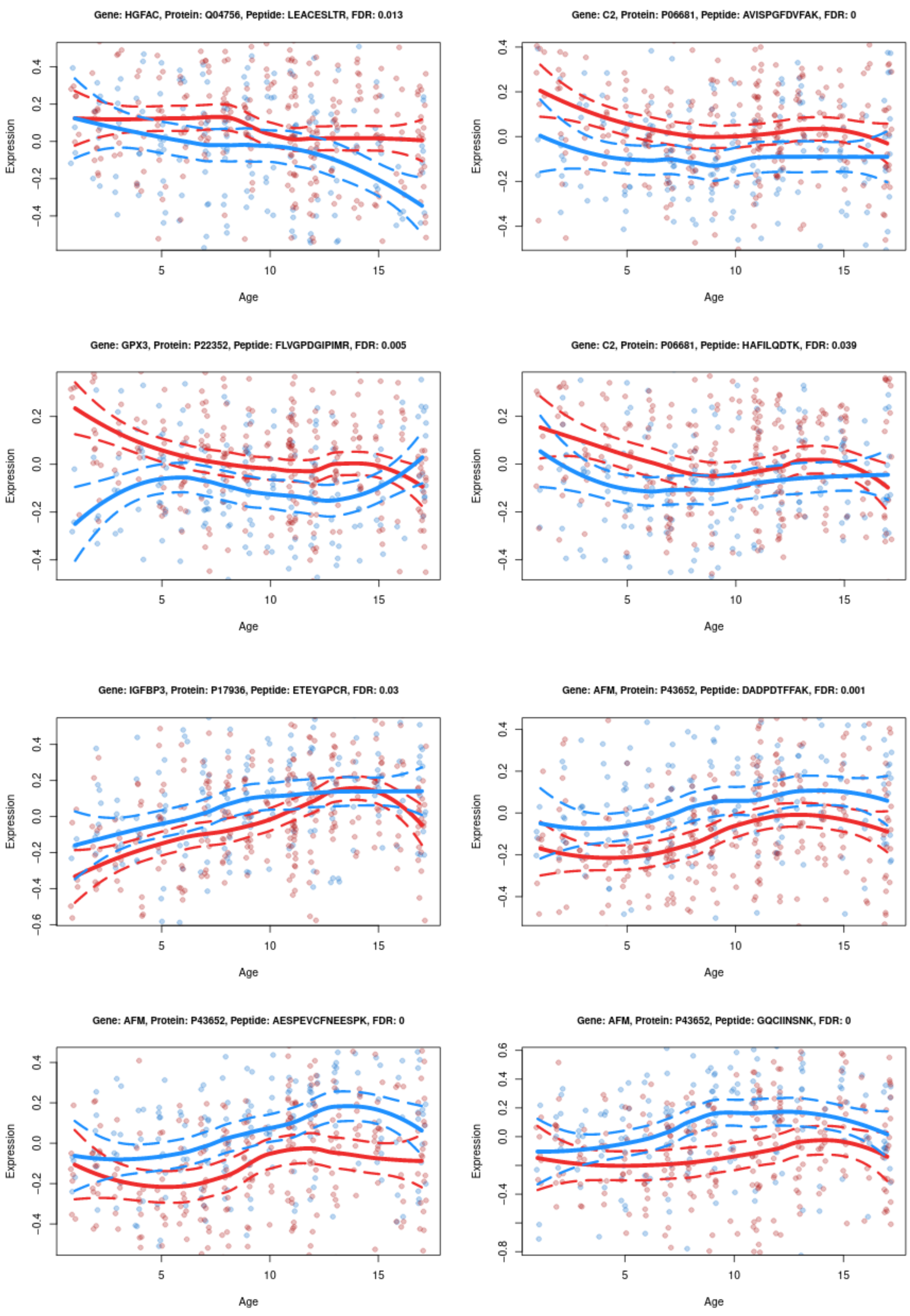

SUPPLEMENTARY INFORMATION

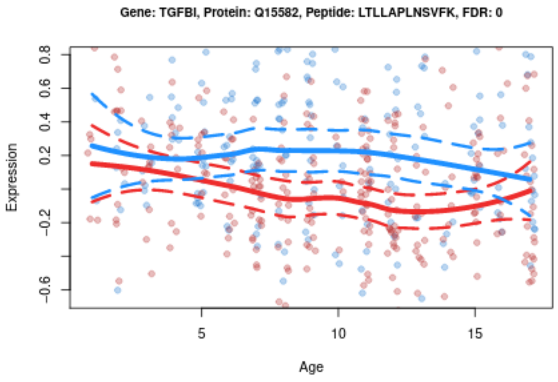

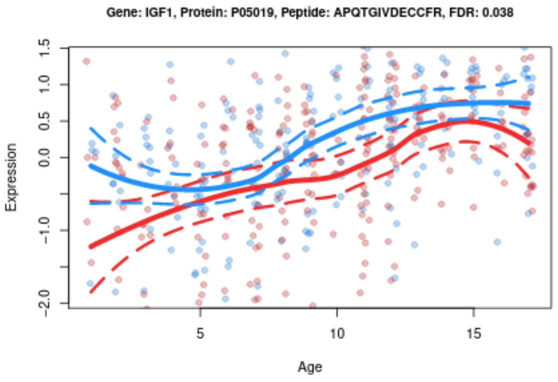

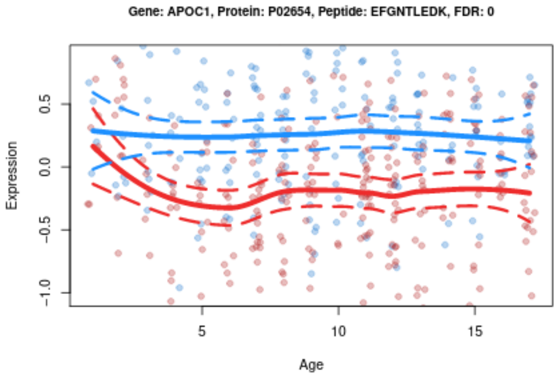

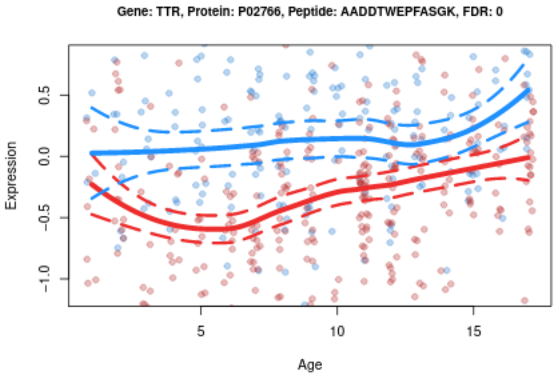

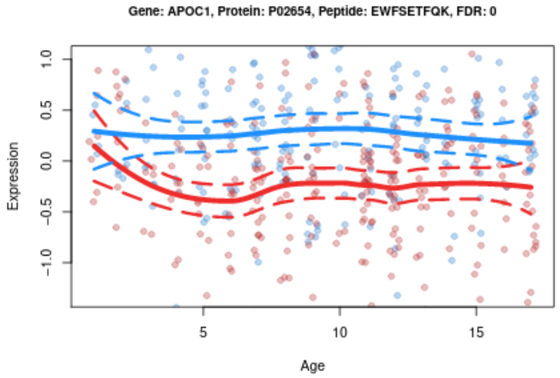

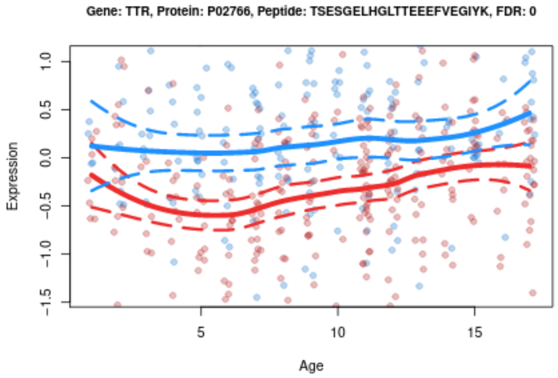

ESM Figure 3: Correlation of the C-peptide/fasting glucose ratio and the mixed meal tolerance test (MMTT) area under the curve (AUC) values. All the available C-peptide / fasting glucose (pmol/l for fasting C-peptide and mmol/l for fasting glucose) values with corresponding MMTT AUC measurements were used from the 3 month, 6 month, 12 month and 24 month visits (n=234 value pairs). The black line displays the linear regression between the two measurements while the blue dashed lines indicate 95% confidence limits for the linear regression predictions.

.

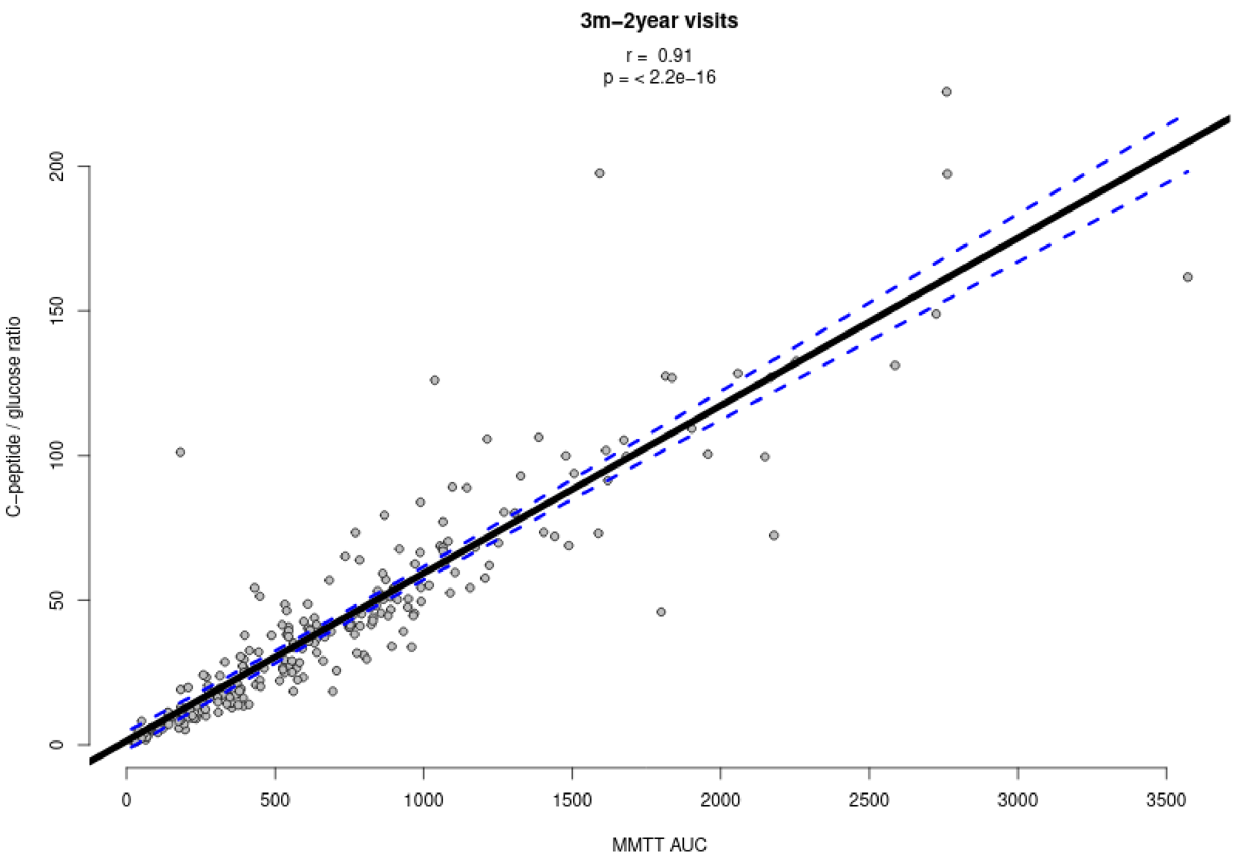

ESM Figure 4: The distribution of Pearson correlation coefficients between the fasting C-peptide/glucose ratio (pmol/l for fasting C-peptide and mmol/l for fasting glucose) and the mixed meal tolerance test (MMTT) area under the curve (AUC) over the 3 month, 6 month, 12 month and 24 month measurements within the newly diagnosed type 1 diabetic individuals of the INNODIA first 100 cohort with enough suitable measurements from both (n individuals = 21).

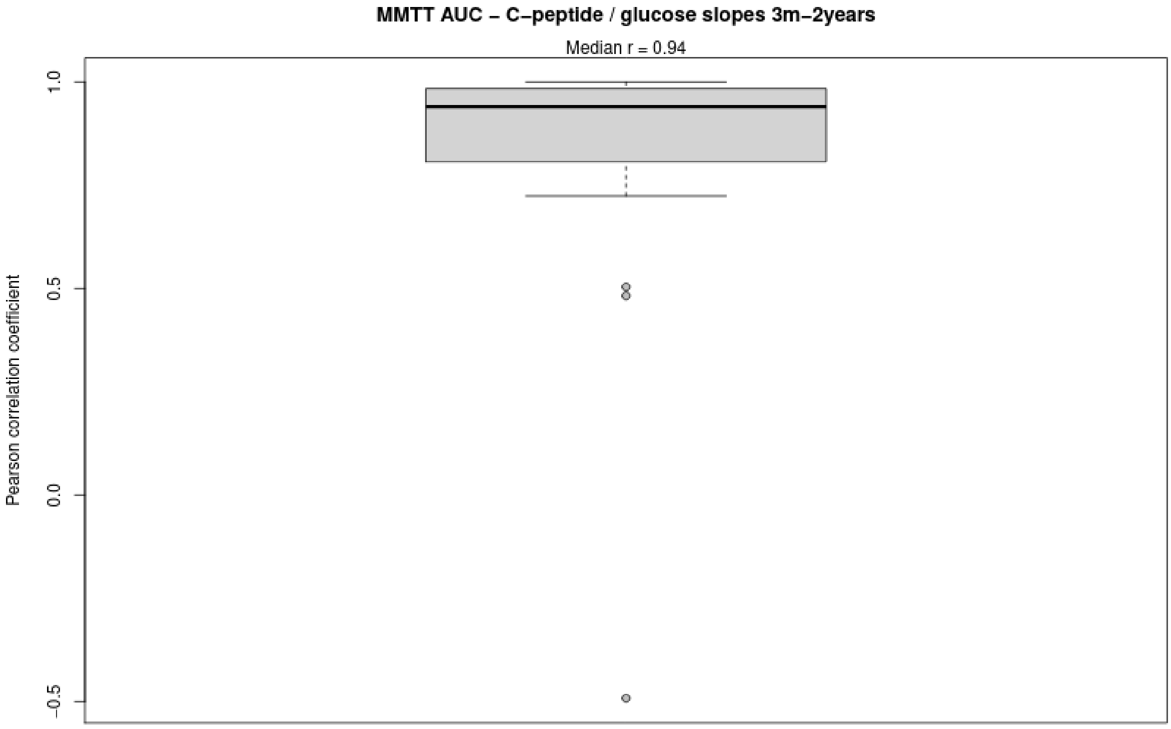

References

1. Metz, T. O. *et al.* Application of proteomics in the discovery of candidate protein biomarkers in a diabetes autoantibody standardization program sample subset. *J. Proteome Res.* **7**, 698–707 (2008).
2. Zhi, W. *et al.* Discovery and validation of serum protein changes in type 1 diabetes patients using high throughput two dimensional liquid chromatography-mass spectrometry and immunoassays. *Mol. Cell. Proteomics* **10**, M111.012203 (2011).
3. Zhang, Q. *et al.* Serum proteomics reveals systemic dysregulation of innate immunity in type 1 diabetes. *J. Exp. Med.* **210**, 191–203 (2013).
4. Moulder, R. *et al.* Serum proteomes distinguish children developing type 1 diabetes in a cohort with HLA-conferred susceptibility. *Diabetes* **64**, 2265–2278 (2015).
5. Liu, C. W. *et al.* Temporal expression profiling of plasma proteins reveals oxidative stress in early stages of Type 1 Diabetes progression. *J. Proteomics* **172**, 100–110 (2018).
6. von Toerne, C. *et al.* Peptide serum markers in islet autoantibody-positive children. *Diabetologia* (2016) doi:10.1007/s00125-016-4150-x [doi].
7. do Nascimento de Oliveira, V. *et al.* Proteomic analysis to identify candidate biomarkers associated with type 1 diabetes. *Diabetes. Metab. Syndr. Obes.* **11**, 289–301 (2018).
8. Geyer, P. E. *et al.* Plasma Proteome Profiling to detect and avoid sample-related biases in biomarker studies. *EMBO Mol. Med.* **11**, e10427 (2019).
9. Albrethsen, J. *et al.* Evaluation of a type 1 diabetes serum cohort by SELDI-TOF MS protein profiling. *Proteomics.Clinical Appl.* **3**, 383–393 (2009).
10. Duchateau, P. N. *et al.* Plasma apolipoprotein L concentrations correlate with plasma triglycerides and cholesterol levels in normolipidemic, hyperlipidemic, and diabetic subjects. *J. Lipid Res.* **41**, 1231–1236 (2000).
11. Lietzen, N. *et al.* Characterization and non-parametric modeling of the developing serum proteome during infancy and early childhood. *Sci. Rep.* **8**, 5883–5885 (2018).
12. Malmström, R. *et al.* Effects of insulin and acipimox on VLDL1 and VLDL2 apolipoprotein B production in normal subjects. *Diabetes* **47**, 779–787 (1998).
13. Bereket, A. *et al.* Effect of insulin on the insulin-like growth factor system in children with new-onset insulin-dependent diabetes mellitus. *J. Clin. Endocrinol. Metab.* **80**, 1312–1317 (1995).
14. Peet, A. *et al.* Circulating IGF1 and IGFBP3 in relation to the development of β-cell autoimmunity in young children. *Eur. J. Endocrinol.* **173**, 129–137 (2015).
15. Jilma, B. *et al.* Elevated circulating P-selectin in insulin dependent diabetes mellitus. *Thromb. Haemost.* **76**, 328–332 (1996).
16. Thrailkill, K. M. *et al.* Matrix metalloproteinase-2 dysregulation in type 1 diabetes. *Diabetes Care* **30**, 2321–2326 (2007).
17. Sapone, A. *et al.* Zonulin upregulation is associated with increased gut permeability in subjects with type 1 diabetes and their relatives. *Diabetes* **55**, 1443–1449 (2006).
18. Jacqueminet, S. *et al.* Elevated circulating levels of matrix metalloproteinase-9 in type 1 diabetic patients with and without retinopathy. *Clin. Chim. Acta.* **367**, 103–107 (2006).
19. Connors, M. H. *et al.* Diminished thyroxine-binding globulin in pubertal diabetic children. *Diabetes Care* **19**, 246–248 (1996).
20. Bhosale, S. D., Moulder, R., Kouvonen, P., Lahesmaa, R. & Goodlett, D. R. Mass Spectrometry-Based Serum Proteomics for Biomarker Discovery and Validation. *Methods Mol. Biol.* **1619**, 451–466 (2017).
21. Bhosale, S. D. *et al.* Serum Proteomic Profiling to Identify Biomarkers of Premature Carotid Atherosclerosis. *Sci. Rep.* **8**, 9209 (2018).
22. MacLean, B. *et al.* Skyline: an open source document editor for creating and analyzing targeted proteomics experiments. *Bioinformatics* **26**, 966–968 (2010).
